# VTR: A Web Tool for Identifying Analogous Contacts on Protein Structures and Their Complexes

**DOI:** 10.3389/fbinf.2021.730350

**Published:** 2021-11-08

**Authors:** Vitor Pimentel, Diego Mariano, Letícia Xavier Silva Cantão, Luana Luiza Bastos, Pedro Fischer, Leonardo Henrique Franca de Lima, Alexandre Victor Fassio, Raquel Cardoso de Melo-Minardi

**Affiliations:** ^1^ Laboratory of Bioinformatics and Systems, Department of Computer Science, Universidade Federal de Minas Gerais, Belo Horizonte, Brazil; ^2^ Laboratory of Molecular Modelling and Bioinformatics (LAMMB), Department of Physical and Biological Sciences, Universidade Federal de São João Del-Rei, Sete Lagoas, Brazil

**Keywords:** protein interactions, structural bioinformatics, structural alignment, contacts, rational design of enzymes

## Abstract

Evolutionarily related proteins can present similar structures but very dissimilar sequences. Hence, understanding the role of the inter-residues contacts for the protein structure has been the target of many studies. Contacts comprise non-covalent interactions, which are essential to stabilize macromolecular structures such as proteins. Here we show VTR, a new method for the detection of analogous contacts in protein pairs. The VTR web tool performs structural alignment between proteins and detects interactions that occur in similar regions. To evaluate our tool, we proposed three case studies: we 1) compared vertebrate myoglobin and truncated invertebrate hemoglobin; 2) analyzed interactions between the spike protein RBD of SARS-CoV-2 and the cell receptor ACE2; and 3) compared a glucose-tolerant and a non-tolerant β-glucosidase enzyme used for biofuel production. The case studies demonstrate the potential of VTR for the understanding of functional similarities between distantly sequence-related proteins, as well as the exploration of important drug targets and rational design of enzymes for industrial applications. We envision VTR as a promising tool for understanding differences and similarities between homologous proteins with similar 3D structures but different sequences. VTR is available at http://bioinfo.dcc.ufmg.br/vtr.

## Introduction

Proteins are macromolecules responsible for most functions in living beings, such as transport, immune protection, control growth, and so on (de Melo et al., 2006). The function of a protein is directly related to its three-dimensional structure. Previous studies have demonstrated that structural changes are related to sequence changes (Chothia and Lesk, 1986). Even small modifications, such as mutations, insertions, or deletions, can change the structure (Almassy and Dickerson, 1978; Lesk and Chothia, 1980; Lesk and Chothia, 1982). However, evolutionarily related proteins may present similar structures but different sequences (Gan et al., 2002). Sequence alignments can unambiguously distinguish similar and non-similar structures when the identity is over 40%. Even sequences with identities of 20–35% may generate false negatives for homology identification (Rost, 1999). Also, studies have reported similarities in structures with sequence identities lower than 20% (Chothia and Lesk, 1986). Until now, the understanding of how the polypeptide sequences fold into a particular three-dimensional shape after synthesis remains a mystery (Science, 2005; Upadhyay, 2019). It has motivated the search for computational algorithms to predict protein structures from their sequences (Dill et al., 2008; Dill and MacCallum, 2012) or even detect and annotate protein functions correctly (Veloso et al., 2007; Franciscani et al., 2014; Silveira et al., 2014). Besides, understanding protein structures and their interactions accurately is crucial to molecule’s rational design for several applications, including discovering novel drugs and improving enzymes for the biotechnological industry (Kuntz, 1992; Barroso et al., 2020).

Thus, understanding the role of inter-residues contacts in protein folding, stabilization, and function has been the goal of several studies (Melo et al., 2007; Silva et al., 2019). Contacts are weak and potentially stabilizing interactions in the structure of macromolecules, such as proteins (Martins et al., 2018; Silva et al., 2019). They can be hydrophobic interactions, electrostatic attractive or repulsive interactions, disulfide bonds, aromatic stacking, hydrogen bonds, and so on Sobolev et al. (1999), Neshich et al. (2003), Mancini et al. (2004), Melo et al. (2007). Contacts also have been used to compare protein structures, for instance, from contact maps (de Melo et al., 2006) or graph-based structural signatures (Pires et al., 2013). Recent computational approaches have suggested that substituting non-interacting residues for interacting ones can improve protein stability, highlighting the importance of computation of contacts (Barroso et al., 2020). In addition, pairwise comparisons between proteins can be performed using visual and structural alignment tools, such as PyMOL (Schrödinger, 2015). However, comparisons between contacts are usually performed individually, which makes comparisons between a considerable number of contacts toilsome. To the best of our knowledge, there is no tool for systematic structural comparisons between contacts in a protein pair.

Therefore, herein, we propose a novel approach to detect and align contacts for protein structure analysis and pairwise comparison. Our algorithm aims to detect differences and similarities in amino acid residues pairs in contact compared to analogous positions. For this purpose, we first perform a structural superposition between two proteins, detect contacts through a cutoff-based approach, and compare contacts in analogous positions using a score. We also developed a user-friendly web tool called VTR to facilitate the use of our method. Finally, as a proof of concept, we propose three case studies: 1) a comparison between a myoglobin (PDB ID: 1a6m) and a hemoglobin (PDB ID: 1dlw), proteins with similar structure but low sequence identity (18%); 2) a comparison of interactions among the spike protein RBD of SARS-CoV-2, SARS-CoV, and the cell receptor ACE2; and 3) a comparison between a glucose-tolerant β-glucosidase enzyme efficient for biofuel production (Bgl1A) and a less efficient non-tolerant β-glucosidase (Bgl1B).

## Materials and Methods

The VTR algorithm receives as input two PDB (Protein Data Bank) (Berman et al., 2000; wwPDB consortium, 2019) files and processes them in the back-end using in-house scripts. The files are analyzed in three steps: 1) structural superposition; 2) contacts computation; and 3) search for contact matches.

### Structural Superposition

VTR performs structural alignments between protein pairs using the default parameters of the TM-align algorithm (Zhang and Skolnick, 2005). TM-align receives as input two PDB files and returns the coordinates of a superimposed structure. TM-align will return the best alignment possible, and VTR will return a warning informing if contact matches could not be found.

### Contact Computation

We compute the Euclidean distance among all-atom coordinates using in-house Python scripts. The tool identifies five types of possible interactions: hydrophobic, attractive/repulsive, hydrogen bonds, aromatic stacking, and disulfide bonds. A pair of residues are in contact if any atom pair meets the cutoff ranges presented in Table 1.

**TABLE 1:** Cutoff distances for each contact type with cutoff values obtained from Sobolev et al. (1999), Neshich et al. (2003), de Melo et al. (2006), Bickerton et al. (2011), Fassio et al. (2019).

Contact type	Atom classes (residue 1–2)	Cutoff (min-max)
Hydrophobic	Hydrophobic - Hydrophobic	2 - 4.5 Å
Attractive/repulsive	Positively - negatively charged (attractive)	2 - 6 Å
	Positively - positively charged (repulsive)	
	Negatively - negatively charged (repulsive)	
Hydrogen bonds	Acceptor - Donor	≤3.9Å
Aromatic stacking	Ring centroid - ring centroid	2 - 6 Å
Disulfide bonds	CYS - CYS	1.5 - 2.8 Å

Contact must occur between atoms of two different residues. The atoms involved are described in Supplementary Table S1. For the detection of aromatic stacking, we determined the centroids of the rings (cutoff 2–6 Å). For phenylalanine and tyrosine, we calculate the median coordinate between the atoms CG and CZ for determining the ring centroid. For tryptophan, we used the median coordinate between the atoms CD2 and CE2. For histidine, we determined the median coordinate of the atoms CG, ND1, CE1, NE2, and CD2.

By default, VTR ignores contacts between atoms of the main chain of neighborhood residues (until reaching four positions) to remove contacts that compose the structures of α-helices. However, through the web tool, users can enable the detection of these contacts (increase processing time).

### Search for Contact Matches

We used in-house scripts to compare the contacts in analogous positions. We proposed the AVD (average vector distance) metric to measure the distance between contacts and detect contact matches. AVD is calculated by Eq. 1:

$$\mathrm{AVD}\left(P,Q\right)=\min\left(\frac{D\left(p1,q1\right)+D\left(p2,q2\right)}{2},\frac{D\left(p1,q2\right)+D\left(p2,q1\right)}{2}\right)$$ (1)

where *P* represents the contact between atoms *p1* and *p2* of protein *A*; *Q* represents the contact between the atoms *q1* and *q2* of protein *B*; and *D* is a function that returns the Euclidean distance between atomic coordinates. To detect a contact match between *P* and *Q*, the *AVD(P, Q)* should be the lowest value. We determine the VTR score based on Eq. 2:

$$\mathrm{VTR}\left(A,B\right)=\frac{\sum_{i=\;1}^{n}\mathrm{AV}D_{i}}{\mathrm{Cn}}\frac{\left(d_{A}+d_{B}\right)}{\left(c_{A}+c_{B}\right)}$$ (2)

where *A* and *B* are the proteins analyzed; n is the number of matches found; *C* is the cutoff determined for the AVD; $d_{A}$ and $d_{B}$ are the number of contacts without match in both proteins; $c_{A}$ and $c_{B}$ are the number of contacts found in each protein. The VTR score is a value between 0 and 1, where the lower the value, the more similar are two proteins in terms of inter-residue contacts.

### Web-Based Tool

The VTR web tool was developed using CodeIgniter (https://codeigniter.com/), D3 (https://d3js.org), jQuery (https://jquery.com), DataTables (https://datatables.net), and Bootstrap CSS and JavaScript library (https://getbootstrap.com). 3D structure visualizations are presented using 3Dmol.js (Rego and Koes, 2015).

### Case Studies Details

For case study 1, we collected the PDBs entries of sperm whale myoglobin (PDB ID: 1a6m) (Vojtechovský et al., 1999) and the *Paramecium caudatum* single-chain and truncated hemoglobin (PDB ID: 1dlw) (Pesce et al., 2000). For the second case study, we selected SARS-CoV-2 (PDB ID: 6m0j) (Lan et al., 2020) and SARS-CoV (PDB ID: 2ajf) (Li et al., 2005) structures in the RCSB PDB. Each PDB file contains two chains A and E, where A represents the cell receptor ACE2 and E the receptor-binding domain (RBD) portion of the virus.

For the third case study, we selected the sequences of the glucose-tolerant GH1 β-glucosidase of a South China Sea metagenome (Bgl1A; UniProt ID: D5KX75) (Fang et al., 2010) and the non-tolerant GH1 β-glucosidase of a South China Sea metagenome (Bgl1B; UniProt ID: D0VEC8) (Fang et al., 2009) from Glutantbase (Mariano et al., 2020). We also constructed two mutants, H57D (Bgl1A) and D57H (Bgl1B), to evaluate VTR’s ability to propose mutations for enzymes based on differences of contacts (Supplementary Tables S5–S6). The 3D structures of wild (Bgl1A and Bgl1B) and mutant (Bgl1A: H57D and Bgl1B: D57H) structures were constructed using SWISS-MODEL (Arnold et al., 2006; Biasini et al., 2014) (Supplementary Tables S7–S8). To evaluate the impact of mutations, we estimated the Poisson-Boltzmann surface area (PBSA) with the integrated use of the online versions of the H++, PDB2PQR, and the Adaptive Poisson-Boltzmann Solver (APBS) tools (Baker et al., 2001; Dolinsky et al., 2004; Unni et al., 2011; Anandakrishnan et al., 2012; Jurrus et al., 2018). Details were included in the supplementary material (Supplementary Text S1).

## Results and Discussion

### VTR Web Tool Workflow

VTR receives as input two PDB files (hereafter called A and B). We suggest that PDBs present similar folding, but if structures with different folds were used, VTR would try to perform the best structural alignment using the TM-align tool. It rotates and translates the coordinates of the protein A considering the alignment with B. VTR allows three search methods: 1) ALL, which calculates all interactions for both whole complexes; 2) SINGLE, which calculates contacts in a single chain for each protein and compares them (users must inform a target chain for each protein); and 3) PPI, which calculates protein–protein interactions in both complexes and then compares them (users must inform a chain pair interacting for each protein).

**FIGURE 1:**
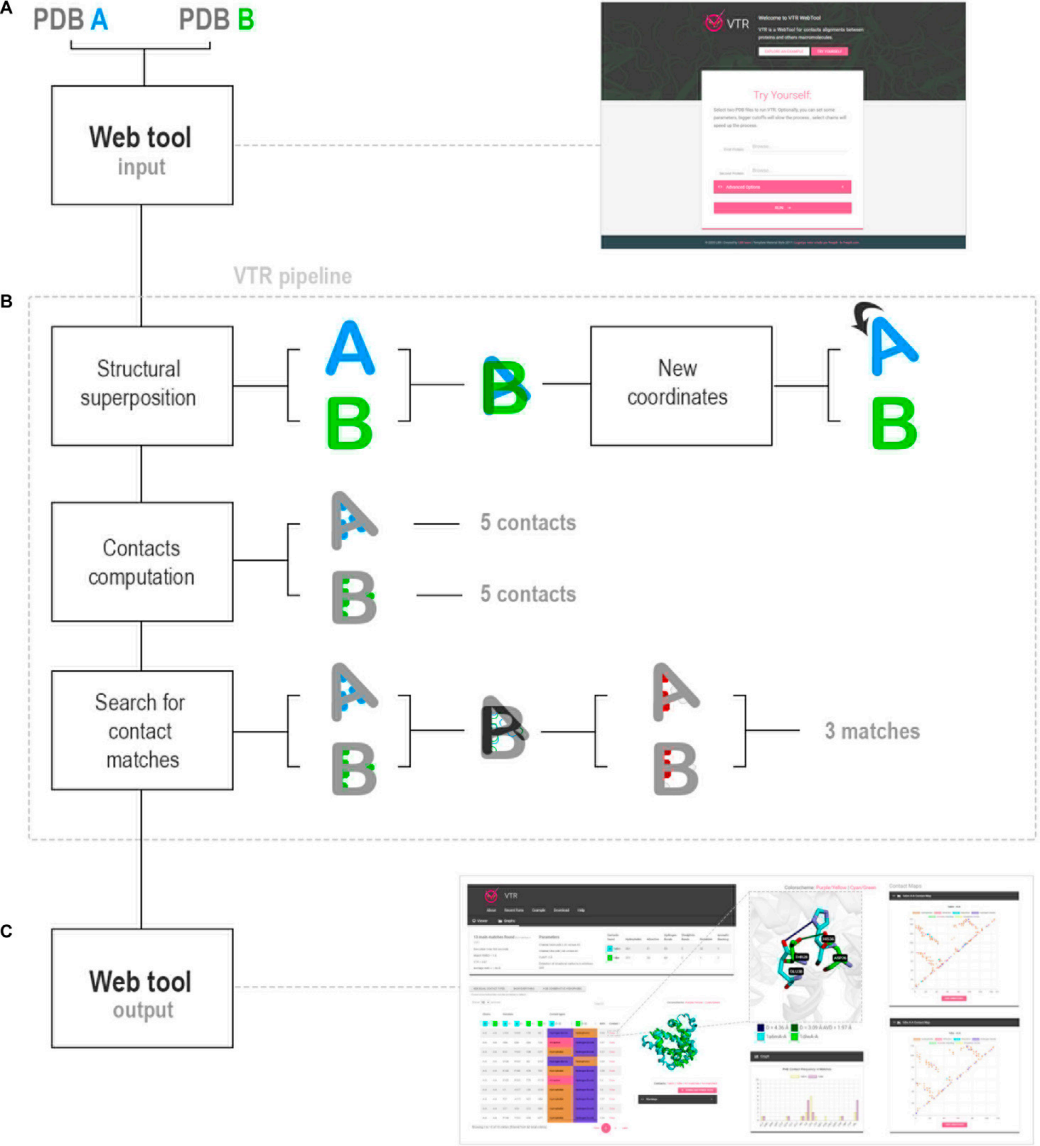
VTR workflow. **(A)** VTR web tool receives as input two PDB files. **(B)** VTR pipeline analyzes the PDB files in three steps: 1) structural superposition between the PDB files using TM-align; 2) contacts calculation using cutoff definitions obtained from the literature; and 3) search for analogous contacts using AVD strategy. **(C)** VTR returns the contact matches, and users can use them in the web interface. Also, VTR determines the statistics of differences between amino acids in contact.

Then, VTR uses in-house Python scripts to calculate contacts: 1) hydrogen bonds; 2) salt bridge (hydrogen bonds and attractive); 3) ionic interaction (attractive); 4) repulsive; 5) hydrophobic interactions; 6) aromatic stacking; and 7) disulfide bonds. Then, VTR determines contacts in analogous positions using the AVD score (average distance between the coordinates of atoms in contact), performs comparative statistical, and returns the results for the VTR interface (Figures 1A–C).

### Case Studies

**FIGURE 2:**
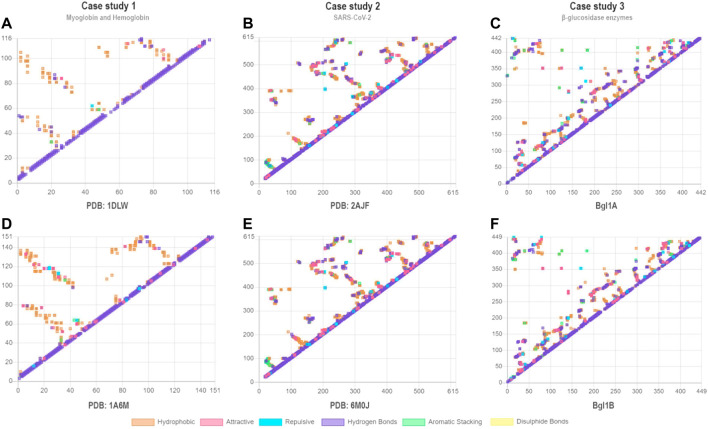
Contact maps for case studies 1 **(A–B)**, 2 **(C–D)**, and 3 **(E–F)**. Each point represents a contact. Contacts of similar types are shown in the same colors. In the x-axes and y-axes are shown the residue numbers. **(A)** PDB ID: 1DLW; **(B)** PDB ID: 1A6M; **(C)** PDB ID: 2AJF; **(D)** PDB ID: 6M0J; **(E)** Bgl1A; **(F)** Bgl1B. For **(C)** and **(D)**, we show only contact maps for chain **(A)**. We consider all contacts for the determination of the contact maps, including contacts between atoms of the main chain of closer residues (such as those present in alpha-helices).

To evaluate the VTR tool, we proposed three pairwise comparative case studies: 1) eukaryotic myoglobin and a truncated non-vertebrate hemoglobin chain; 2) interactions among the cell receptor ACE2 and both SARS-CoV-2 and SARS-CoV; and 3) glucose-tolerant and non-tolerant β-glucosidases. Contact maps generated by VTR demonstrate similarities in the contact pattern between protein pairs in each case study. For instance, Figure 2A is more similar to Figures 2B–F. We aim with these case studies to demonstrate a simple use for VTR (case study 1) and show the tool’s effectiveness in finding shared and unshared contacts in two systems already described in the literature (case study 2). In the third case study, we propose a real use of VTR in detecting possible mutation sites for enhancing enzymes with biotechnological applications.

#### Case Study 1: Myoglobin and Hemoglobin

In the first case study, we performed the contacts alignment between sperm whale myoglobin (PDB ID: 1a6m) and truncated single-chain hemoglobin from *Paramecium caudatum* ciliated protozoan (PDB ID: 1dlw). Myoglobin and hemoglobin are both oxygen-binding proteins belonging to the widespread and distantly related globin family (Hardison, 2012). For this case study, the evaluated myoglobin structure (1a6m) was at the oxy state (i.e., oxygen bound), while the hemoglobin structure (1dlw) was at the de-oxy one (i.e., oxygen unbound). The 1a6m presents a sequence length of 151 amino acids, while 1dlw, as a typical non-vertebrate truncated hemoglobin chain, has a minor amino acid content, with just 116 residues. Both proteins present a similar folding but a sequence identity of only 18%. The literature has described those structures of homologous sequences with an identity lower than 20% may present large structural differences (Chothia and Lesk, 1986). However, the discrepancy about this typical strong relationship between sequence and folding similarity for the globin family has long been known. In fact, globins form a family substantially conserved in structural topology, despite the distant sequence relationship, being this one of the higher conundrums in biochemistry (Lesk and Chothia, 1980; Hardison, 2012). Hence, we believe these proteins to be potential targets for the comparison of analogous contacts using VTR.

After processing both structures with default parameters, VTR detected the following contacts for 1a6m: 85 hydrogen bonds, 81 attractive interactions, 32 repulsive interactions, nine aromatic stacking, and 364 hydrophobic interactions. We found the following contacts in 1dlw: 65 hydrogen bonds, 20 attractive interactions, one repulsive interaction, two aromatic stacking, and 221 hydrophobic interactions. However, we obtained only 13 main contact matches in analogous positions using a 2 Å AVD cutoff (Supplementary Table S2). The contact matches increase to 60 when considering conserved contact matches of hydrophobic type.

**FIGURE 3:**
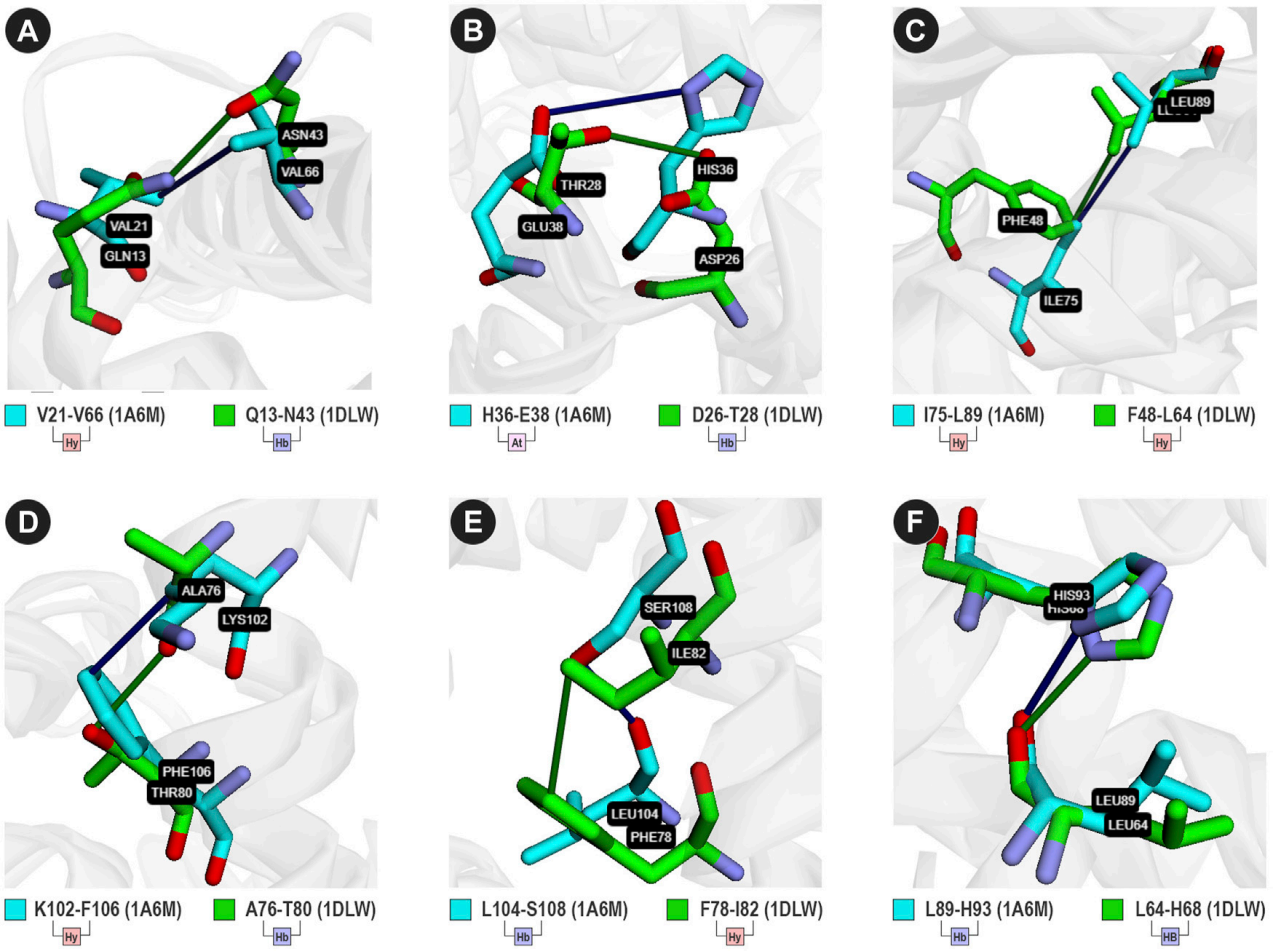
Six analogous contacts between 1a6m (cyan sticks) and 1dlw (green sticks). (Hy) hydrophobic; (Hb) Hydrogen bond; (At) Attractive ionic interaction. **(A)** V21-V66 (1a6m) and Q13-N43 (1dlw); **(B)** H36-E38 (1a6m) and D26-T28 (1dlw); **(C)** I75-L89 (1dlw) and F48-L64 (1dlw); **(D)** K102-F106 (1a1m) and A76-T80 (1dlw); **(E)** L104-S108 (1a6m) and F78-I82 (1dlw); and **(F)** L89-H93 (1a6m) and L64-H68 (1dlw). Sticks were colored using the cyan/green color scheme.

From the contacts predicted in analogous positions, we found 12 different types (Figure 3; Supplementary Tables S2–S4), such as V21-V26 and Q13-N43 (Figure 3A). For 1a6m, VTR detected an attractive contact between H36 and E38. However, it detected a hydrogen bond between D26 and T28 in analogous positions of 1dlw (Figure 3B). On the other hand, in 1a6m, I75 performs hydrophobic interactions with L86, while F48 performs the same type of interaction with V109 (Figure 3C). Interestingly, I75 and L86 are located at a distance of 11 amino acids, while F48 and V109 are at a distance of 61 positions. This highlights the VTR’s ability to detect contacts even in different sequence positions. Most of the contact matches are located at compatible distances of 2–4 amino acids, such as K102-F106 and A76-T80 (Figure 3D), L104-S108 and F78-I82 (Figure 3E), and L89-H93 and L64-H68 (Figure 3F). Only the hydrogen bond detected in the contact L89-H93 of 1a6m was considered a match with Q13-N43. This may suggest that both contacts present similar importance for the stability of both proteins.

It is essential to highlight that, for this analysis, we did not consider the interactions between atoms of the main chain of closer residues. We did it to reduce the number of contacts detected in alpha-helices, which can explain the low number of hydrogen bonds conserved. Enabling the advanced option “detection of structural contacts in α-helices,” the number of hydrogen bond contact matches increases to 129. This option is disabled by default because VTR desires to highlight conserved interactions with the side-chain atoms. Also, enabling this option increases the computational cost once that all contacts of similar secondary structures in close regions will be computed.

#### Case Study 2: Analyzing Interactions Between the Spike Protein RBD of SARS-CoV-2 and the Cell Receptor ACE2

Recently, a new coronavirus (SARS-CoV-2) was related to severe acute respiratory syndrome (COVID-19), spreading rapidly worldwide, and causing a pandemic situation (Zhou et al., 2020). A sequence comparison demonstrates that SARS-CoV-2 structures share approximately 80% of sequence identity with the SARS-CoV (Lan et al., 2020; Zhou et al., 2020). Like SARS-CoV, SARS-CoV2 recognizes the ACE2 (Angiotensin-Converting Enzyme 2) receptor in humans. Hence, understanding the binding mechanism of the spike RBD (Receptor-Binding Domain) of SARS-CoV-2 with ACE2 may help to shed some light on the mechanism of recognition of virus receptors and the initial infection process. In a recent study, Lan et al. (2020) identified the relevant residues to the interaction of SARS-CoV-2 with the receptor. It was noted that most of these residues are highly conserved and shared with SARS-CoV. Here, we verified the ability of VTR to find the known contacts between different chains of both structures. We compared the structures of SARS-CoV-2 spike RBD (PDB ID 6m0j) and SARS-CoV spike RBD (PDB ID 2ajf), both in complex with the ACE2 receptor. We maintain the standard 2 Å AVD cutoff, and we use chain A (ACE2) and chain E (RBD portion of the virus) for both structures.

Compared to a study by Lan et al. (2020), the VTR could find all 16 atomic contacts between SARS-CoV and ACE2 (3 salt bridges and 13 hydrogen bonds). VTR could also find 15 contacts between SARS-CoV-2 and ACE2 (2 salt bridges and 13 hydrogen bonds). Of these, VTR calculated and presented which contacts were shared between receptor and SARS-CoV or SARS-CoV-2. We detected all contacts described in Lan et al. (2020) (Table 2).

**TABLE 2:** Analogous contacts between proteins with PDB entries SARS-CoV (6m0j) and 2ajf. R1: residue 1; R2: residue 2; HB: hydrogen bonds, HY: hydrophobic. In the last column, we highlighted lines where the matches were reported in Lan et al. (2020). Residues in contact, but atom interactions were not described in Lan et al. (2020), are presented as “−.”

	Chain: Residue (atom)				Contact type		Reported in (Lan et al., 2020)
	SARS-CoV-2 (R1)	SARS-CoV-2 (R2)	SARS-CoV (R1)	SARS-CoV (R2)	SARS-CoV-2 R1-R2	SARS-CoV R1-R2	
1	A:Y41 (OH)	E:N501 (N)	A:Y41 (OH)	E:T487 (N)	HB	HB	✓
2	A:Y41 (OH)	E:N501 (OD1)	A:Y41 (CE2)	E:T487 (CG2)	HB	*HY*	—
3	A:D38 (OD2)	E:Y449 (OH)	A: D38 (OD2)	E:Y436 (OH)	HB	HB	✓
4	A:Y83 (OH)	E:N487 (OD1)	A:Y83 (OH)	E:N473 (ND2)	HB	HB	✓
5	A:H34 (NE2)	E:Y453 (OH)	A:H34 (NE2)	E:Y440 (OH)	HB	HB	—
6	A:E37 (OE2)	E:Y505 (OH)	A:E37 (OE1)	E:Y491 (OH)	HB	HB	—
7	A:D38 (OD1)	E:Y449 (OH)	A:D38 (OD1)	E:Y436 (OH)	HB	HB	—
8	A:G354 (O)	E:G502 (N)	A:G354 (O)	E:G488 (N)	HB	HB	—
9	A:F28 (N)	E:Y489 (OH)	A:F28 (N)	E:Y475 (OH)	HB	HB	—
10	A:Y83 (OH)	E:Y489 (OH)	A:Y83 (OH)	E:Y475 (OH)	HB	HB	✓
11	A:Q42 (NE2)	E:Y449 (OH)	A:Q42 (OE1)	E:Y436 (OH)	HB	HB	—
12	A:Y41 (OH)	E:T500 (OG1)	A:Y41 (OH)	E:T486 (OG1)	HB	HB	✓
13	A:Q24 (OE1)	E:N487 (ND2)	A:Q24 (OE1)	E:N473 (ND2)	HB	HB	✓
14	A:K353 (O)	E:G502 (N)	A:K353 (O)	E:G488 (N)	HB	HB	✓

**FIGURE 4:**
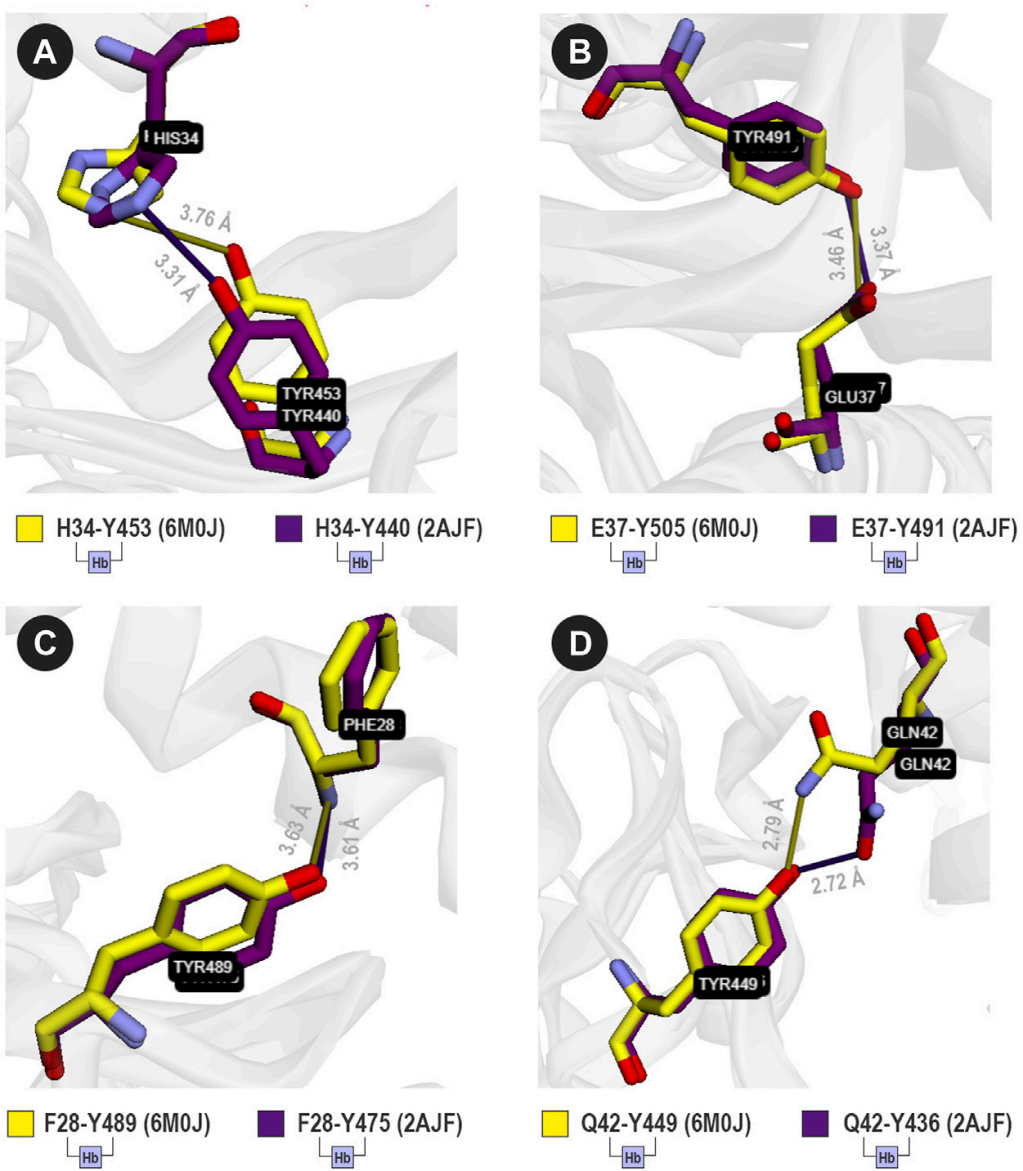
Four analogous contacts between SARS-CoV and ACE2 (purple sticks), and SARS-CoV-2 and ACE2 (yellow sticks). VTR suggests that **(A)** H34-Y453 (6M0J) is equivalent to H34-Y440 (2AJF); **(B)** E37-Y505 (6M0J) is equivalent to E37-Y491 (2AJF); **(C)** F28-Y489 (6M0J) is equivalent to F28-Y475 (2AJF); and **(D)** Q42-Y449 (6M0J) is equivalent to Q42-Y436 (2AJF). Sticks were colored using the yellow/purple color scheme.

VTR calculated six shared hydrogen bonds and one hydrophobic interaction between SARS-CoV and a receptor that are not present in Lan et al. (2020) (Table 2). Although the hydrophobic interaction between Y41 (CE2) and T487 (CG2) was not described in Lan et al. (2020), the hydrogen bond between Y41 and T487 was presented (Table 2; lines 1 and 2). VTR also detected a permuted interaction between atoms of D38 and Y449 (6m0j) and D38 and Y436 (2ajf) (Table 2; lines 3 and 7). Moreover, the interactions between G354 (O) and G502 (N) of 6m0j and their equivalents in 2ajf appear to be probably detected due to the cutoff-based strategy (Table 2; line 8). Also, VTR detected that G502 interacted with K353: interaction described in Lan et al. (2020). Among the conserved hydrogen bond contacts, four called our attention as they were described in the literature (Figures 4A–D).

For SARS-CoV-2, VTR detected a hydrogen bond between H34 and Y453 that seems to be equivalent to H34 and Y440 in SARS-CoV (Figure 4A). The same applies to the following contact pairs: E37-Y505 (6M0J) and E37-Y491 (2AJF) (Figure 4B); F28-Y489 (6M0J) and F28-436 (2AJF) (Figure 4C); and Q42-Y449 (6M0J) and Q42-Y436 (2AJF) (Figure 4D). It is important to highlight that besides the interaction between E37 and Y505 (Figure 4B), the residue E37 possibly interacts with R403 in 6M0J, but there is no equivalent contact in 2AJF. Also, Lan et al. (2020) described an interaction between Y83 (OH) and Y489 (OH) of 6M0J. Besides this interaction, VTR also pointed out that Y489 (OH) interacts with the main-chain atoms of F28. It demonstrates that the cutoff-based strategy used by VTR also has advantages when compared to more restrictive methods. This possible interaction could, in future studies, be better comprehended through molecular dynamics experiments.

#### Case Study 3 - Glucose-Tolerant β-glucosidases

β-glucosidases (E.C. 3.2.1.21) are enzymes that act in the last step of the saccharification process, cleaving cellobiose into two molecules of glucose (Ketudat Cairns and Esen, 2010; Mariano et al., 2017). Hence, they are considered very important for the second-generation biofuel industrial applications (Bergmann et al., 2014; Costa et al., 2019; Limade et al., 2020). Besides, the literature has reported that most β-glucosidases are inhibited in high glucose concentrations (Teugjas and Väljamäe, 2013; de Giuseppe et al., 2014). Therefore, the design of enzymes more resistant to glucose inhibition has motivated research around the world (Salgado et al., 2018). Recently, two β-glucosidases extracted from the marine metagenome of the South China Sea were characterized and evaluated (Yang et al., 2015). The first one (Bgl1A) was able to keep its activity even in glucose concentrations up to 1,000 mM (Fang et al., 2010), being described as glucose-tolerant (a class of β-glucosidase enzymes with high potential for industrial use). On the other hand, the second one (Bgl1B) was inhibited in concentrations up to 50 mM (Fang et al., 2009). Both enzymes have a similar TIM-barrel folding, belonging to the first family of glycoside hydrolases (GH1) (Cantarel et al., 2009). Moreover, they present a sequence similarity higher than 50% (Mariano et al., 2019). Thus, we decided to submit to VTR the structures of Bgl1A and Bgl1B to evaluate similar contacts and identify possible differences.

VTR found 375 main contact matches (984 considering hydrophobics), an average RMSD (for contact matches) of 0.96, and a VTR score of 0.19. From 375 matches, 327 maintain the contact type, and 48 change the contact type. The matches that change the contact type are interesting targets for the detection of differences in structures and potentially can be used to suggest mutations. Thus, such information may guide a biotechnological industry at providing glucose tolerance characteristics to Bgl1B through the rational design of enzymes using site-directed mutagenesis.

**FIGURE 5:**
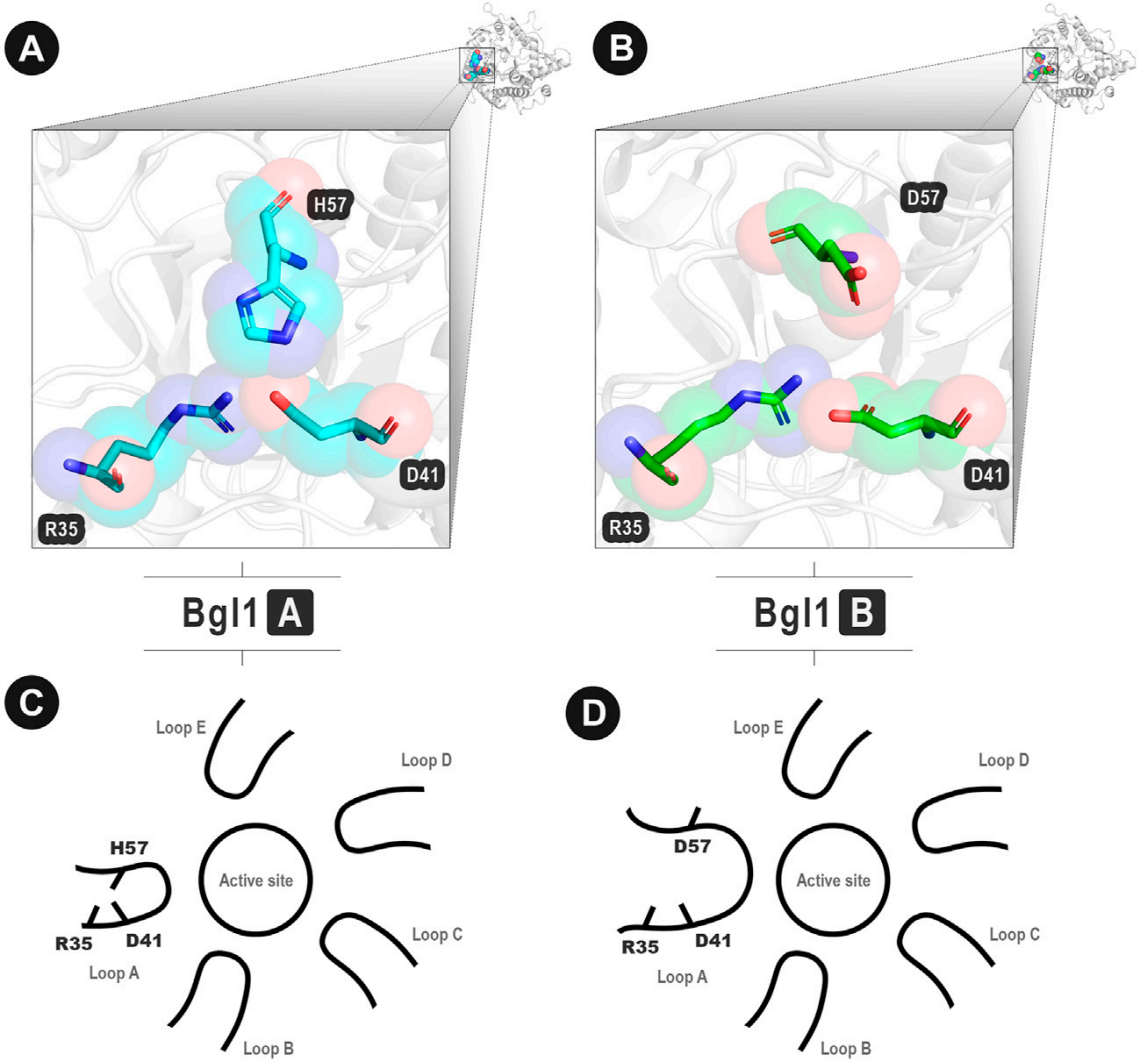
Important contacts in analogous positions between Bgl1A and Bgl1B. **(A)** Attractive interaction D41-H57 in loop A of Bgl1A. **(B)** Repulsive interaction D41-D57 in loop A of Bgl1B. **(A–B)** Protein backbones are shown as grey cartoons. Amino acid residues are shown as cyan (Bgl1A) and green (Bgl1B) cartoons. In both proteins, D41 also interacts with R35. **(C–D)** Illustrative scheme of the importance of these contacts. **(C)** Bgl1A: H57 performs attractive interactions with D41. **(D)** Bgl1B: D41 performs repulsive interactions with D57.

After analyzing the results (available in the Supplementary material), one contact match caught our attention: D41-H57 of Bgl1A (Figure 5A) that corresponds to D41-D57 of Bgl1B (Figure 5B). While Bgl1A presents an attractive contact, Bgl1b presents a repulsive interaction. These contacts are located in the extremities of loop A. Furthermore, this loop has been reported in the literature as necessary for restricting the entrance and exit of the active site in glucose-tolerant β-glucosidases (Limade et al., 2020). Hence, changes in this region could modify the mobility of the protein and could explain the differences in the glucose tolerance of both enzymes.

The literature has reported implications of the topology and dynamics of loop A for the differences in glucose tolerance and inhibition between Bgl1A and Bgl1B (Yang et al., 2015; Limade et al., 2020). To probe the potential of our method for protein engineering, we have checked how considerable were the physical-chemical differences attributed by this point modification at loop A as a whole.

Firstly, we modeled *in silico* a mutant of Bgl1A (D57H) and a mutant of Bgl1B (H57D). We expected that Bgl1B’s mutant presented characteristics similar to Bgl1A (*i.e.*, characteristics that could lead to glucose tolerance). As a control, we modeled a Bgl1A mutant that we expected to present features like Bgl1B (*i.e.,* negative characteristics for biofuel production). Then, we inspected the mesoscopic influence of such single amino acid modification at the regional electrostatic surface by Poisson-Boltzmann analysis.

##### PBSA Points Substantial Electrostatic Differences

**FIGURE 6:**
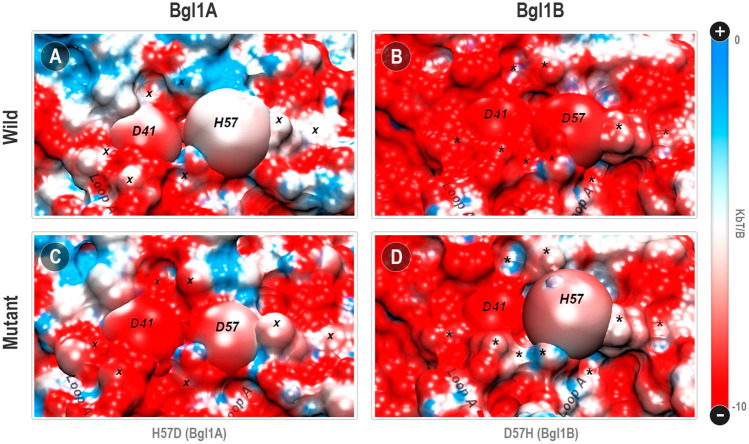
Poisson-Boltzmann surface potential at the vicinity between positions 41 and 57 in Bgl1A, Bgl1B, and mutants. **(A)** Bg1lA; **(B)** Bgl1B; **(C)** Bgl1A’s H57D mutant; **(D)** Bgl1B’s D57H mutant. The PBSA colors are on a red: white: blue scale for electrostatic potentials around −10.00: −5.00: 0.00 k_b_T/*β* unities (being k_b_ the Boltzmann constant, T the 300 K temperature, and *β* the electron coulomb charge). Residues 41 and 57 are depicted as spheres. Regions whose electrostatic potential is affected by the H57D or D57D permutations are depicted as “x” for Bgl1A or “*” for Bgl1B.

The estimation of the protonation states at pH 7.0 has recovered the usual HSE protonation for the H57 in Bgl1A (*i.e.*, a neutral histidine protonated just at the *ε* nitrogen atom). This is consonant with the position of the side chain of this histidine in loop A relatively turned to the solvent, with just a marginal contact with the D41 residue at the opposite side. Also, the presence of the positively charged R35 prevents that the H57 local pKa be changed enough by the next D41 to induce a bi-protonation at this histidine (Figure 5). Hence, while the D41-D57 in Bgl1B can be classified as an electrically repulsive contact, the D41-H57 interaction in Bgl1A is not a charge to charge but a charge-dipole attractive interaction. Even so, the Poisson-Boltzmann surface analysis (PBSA) indicates a substantial difference between Bgl1A and Bgl1B in the electrostatic surface potential of loop A (Figures 6A,B).

Indeed, all the topological context around loop A in Bgl1B accumulates a substantial amount of negative electrical potential (red region in Figure 6B). On the other hand, Bgl1A presents a surface with a more neutral electrical potential (blue and white regions in Figure 6A). While the D41-D57 contact appears to be a hot spot of negative charge accumulation in Bgl1B, the correspondent D41-H57 in Bgl1A shares similar neutrality of the surrounding region. The substitutions H57D (Bgl1A) and D57H (Bgl1B) introduce a point inversion of this behavior in both proteins (Figures 6C,D). Also, these permutations cause electrostatic modifications at specific points of this region. Comparing Figures 6A,C (Bgl1A and its mutant), we can observe an increase of red regions. Contrarily, comparing Figures 6B,D (Bgl1B and its mutant), we can observe a reduction of the red regions.

Furthermore, the sites with local potential affected by the permutations appear to be the same in Bgl1A and Bgl1B, spread around loop A. The dynamics and topology of this region are strongly correlated to the functional differences between both proteins (Yang et al., 2015; Limade et al., 2020). In addition, the charge inversions in this region have been correlated to changes in activity and stability (González-Blasco et al., 2000; Tamaki et al., 2014). All these factors led us to look for a glimpse of the influence of the D41-H57D permutation in the vibrational dynamics of loop A.

##### Vibrational Modulation of Loop A

The representativity of the eigenvectors (Supplementary Figure S1) demonstrates the differences between the PCA recovered fluctuation between loops A of Bgl1A and Bgl1B. Bgl1A fluctuation is more homogeneously represented by the two first eigenvectors (PCs) of loop A, with a fractional eigenvalue of 63% for PC1 and 29% for PC2. On the other hand, the fluctuations at the Bgl1B’s loop A are strongly located at PC1, that alone accounts for 82% of the sum of all eigenvalues. This can indicate that a single kind of movement, with a substantial fugacity from the middle structure at the minima, accounts for most of the vibratory mobility of Bgl1B’s loop A. This is consonant with a local instability caused by the highly repulsive environment at this loop (Figure 6B).

In Supplementary Figure S1, we can see higher proximity among the lines that represent wild Bgl1B, Bgl1B’s mutant, and Bgl1A’s mutant. The loop A of the wild Bgl1A presents a more equilibrated and distributed fluctuation between different modes (in concurrence with the more neutral profile in Figure 6A). In addition, while the H57D exchange is enough to invert the vibrational eigenvalue distribution of the Bgl1A’s loop A to the Bgl1B pattern, the same does not occur for Bgl1B with the D57H permutation (Supplementary Figure S1A). Hence, the more neutralized electrostatic surface for Bgl1A (Supplementary Figure S1A) is sensitive to the point insertion of a repulsive contact at the loop A basis. On the other hand, the strongly negatively charged Bgl1B’s loop A is less responsive to neutralization at this single point.

Furthermore, the analysis of the distribution of the fluctuations allocated at the two first eigenvectors (PC1+2) of loop A also shows considerable differences (Supplementary Figures S1B–F). Bgl1A presents higher apparent vibrational mobility concentrated at the C-terminal portion of the N-terminal helix of this loop (residues 45–50). In contrast, Bgl1B has its high mobility equally spread along the entire medial portion of loop A (residues 45–55). The different electrostatic contexts (Figures 6A,B) may explain the vibrational distinctions (Supplementary Figures S1A–B). Despite this, the impact in contact patterns, caused by changes in position 57, promotes at least qualitatively standardized modifications around half of loop A (region indicated by lines in Supplementary Figures S1C–F). The presence of a negatively charged amino acid in position 57 promotes, both in Bgl1A and Bgl1B’s environments, an increase in mobility of positions 44, 50, 51, and 57 itself. It also promotes a reduction in the mobility of the region between residues 45 to 49.

For the D41-D57 repulsive contact, the vibrational movement of both acids is unbalanced, with one moving more or faster than the other (“*” in Supplementary Figures S1D–E). On the other hand, the movement involving the D41-H57 contact is more balanced (mainly for Bgl1A’s context), indicating a more equilibrated vibration again.

##### Implications for Protein Engineering

The intensity of some of these modifications or changes at the rest of loop A seems to be context-dependent. This agrees with the fact that the permutation of the entire loop A between Bgl1A and Bgl1B was able to revert the glucose tolerant/inhibited behavior, but with poor results for local single amino acid substitutions (Yang et al., 2015). Despite this, a single substitution detected by VTR was able to promote vibrational changes with the same pattern at almost half of the extension of this functionally crucial loop.

All these mentioned facts illustrate a potential use for this tool. If properly combined with electrostatic and mobility patterns detection techniques, such as the APBS and molecular mechanics/PCA here employed, VTR can be useful for rational protein engineering. The integrated use of VTR with computational or experimental tools can be promising to find the topologically minimum modifications that need to be carried at proteins to introduce some desirable characteristics found in other homologous.

For GH1 β-glucosidases, a protein class extensively used in industry and with a strong interest in the second-generation bioethanol production (Mariano et al., 2017), this approach shows itself to be encouraging. Overall, minimal topological modifications represent a promising strategy for suggesting mutations, especially in the beta-glucosidase loop regions that surround the active site. The topology and dynamics of these loops can allow or restrict movements involved in glucose entrance and exit (*i.e.*, glucose tolerance) (Yang et al., 2015; Costa et al., 2019; Konar et al., 2019; Limade et al., 2020) or also can affect the thermostability (González-Blasco et al., 2000; Tamaki et al., 2014; Konar et al., 2019). These are both examples of industrially desirable characteristics for these and other proteins.

## Conclusion

Herein, we presented VTR, a novel approach with a visual web interface that can be used to analyze, compare, and scrutinize analogous contacts in protein pairs. We explored the tool features using three case studies, where we demonstrated the potential of VTR to shed some light on the mechanisms of topology conservation on phylogenetically related but sequentially distant proteins. We also evaluated contact similarities and dissimilarities on pharmacologically relevant targets. We suggested the use of our tool to the rational design of proteins with biotechnological applications. Concerning the second case study, both the confirmation of contacts already reported in the literature and the finding of four hydrogen bond matches still not described are promising finds for the future rational design of anti-Sars-Cov-2 drugs. In the last case study, we compared a glucose-tolerant beta-glucosidase enzyme with a non-tolerant one. VTR detected several changes in contact types of analogous positions. Called our attention a change in an attractive contact by a repulsive: D41-H57 of Bgl1A that corresponds to D41-D57 of Bgl1B. We explored the importance of this contact using molecular mechanics minimization and vibrational inspection by principal component analysis. Our results demonstrate that the presence of this contact is vital for the stability of Bgl1A. However, Bgl1B’s mutant with a similar interaction was not enough to present similar characteristics of the glucose-tolerant one on a substantial extension. Nevertheless, this case study illustrates the potential of the VTR tool for the rational design of industrial enzymes and gives some glimpses about electrostatic and vibrational aspects of β-glucosidase enzymes. We hope VTR can be used for understanding differences and similarities between homologous proteins with similar 3D structures but differences in sequences. VTR is available at http://bioinfo.dcc.ufmg.br/vtr.

## Data Availability

The original contributions presented in the study are included in the article/Supplementary Material, further inquiries can be directed to the corresponding author.
