# Supplementary material for: VTR: A Web Tool for Identifying Analogous Contacts on Protein Structures and Their Complexes
Source: Front Bioinform. 2021 Nov 8;1:730350. doi: 10.3389/fbinf.2021.730350 (PMC9581016; doi:10.3389/fbinf.2021.730350)
Supplement: Supplementary file 1 [file DataSheet1.PDF]

Supplementary material

for "VTR: a web tool for identifying analogous contacts on protein structures and their complexes"

Vitor Pimentel[1], Diego Mariano[1], Letícia Xavier Silva Cantão[1], Luana Luiza Bastos[1], Pedro Fischer[2], Leonardo Henrique França de Lima[2], Alexandre Victor Fassio[1], Raquel Cardoso de Melo-Minardi[1]*

[1] Laboratory of Bioinformatics and Systems. Department of Computer Science. Universidade Federal de Minas Gerais, Belo Horizonte, 31270-901, Brazil.
[2] Laboratory of Molecular Modelling and Bioinformatics (LAMMB), Department of Physical and Biological Sciences, Universidade Federal de São João Del-Rei, Campus Sete Lagoas, Sete Lagoas, 35701-970, Brazil.
*To whom correspondence should be addressed: RCMM: raquelcm@dcc.ufmg.br; DCBM: diegomariano@ufmg.br

Summary

Additional text S1. Material and methods (case study 3) 2

Figure S1. Vibrational mobility pattern recovered by minimization and PCA for loop A of Bgl1A, Bgl1B, and mutants. 5

Table S1. Atoms classes for each type of contact. Obtained from (Sobolev et al., 1999; Fassio et al., 2019). 7

Table S2. Analogous contacts between proteins with PDB entries 1a6m and 1dlw. 8

Table S3. 1A6M Contacts (Chain A). 9

Table S4. 1DLW Contacts (Chain A). 13

Table S5. Models Assessment (BGL1B). 16

Table S6. Models Assessment (BGL1A) 19

Table S7. Models Assessment (D57H__BGL1B) 21

Table S8. Models Assessment (H57D_BGL1A) 22

References 25

Additional text S1. Material and methods (case study 3)

The online H++ server was used to estimate the protonation states using the default salinity and dielectric parameters (respectively, 0.15 M, the internal dielectric of 10 and 80) and pH 7.00 (Anandakrishnan *et al.*, 2012). Then, the PDB2PQR software was used to generate the input files for the PBSA calculations with APBS. Next, we used APBS to estimate the surface electrostatic potential along the protein surface according to the linearized numerical approximation of the differential Poisson-Boltzmann equation (1):

$$\boldsymbol{\nabla}^2\psi = -\frac{c_0\beta}{\epsilon_{solv}\cdot\epsilon_{sol}}\cdot\left[e^{\frac{-\beta\psi(x,y,z)}{k_BT}} - e^{\frac{\beta\psi(x,y,z)}{k_BT}}\right] \quad (1)$$

where Ψ is the electric tridimensional potential along the solute surface; *c0* is the solvent ionic concentration (0.15 M); ε_{solv} and ε_{sol} are the respective dielectric values of the solvent (78.54; default value) and of the solute (2; default value); β is the absolute value of the charge of an electron (1.602 x 10^{-19} coulombs), T is the absolute temperature in Kelvin and k_B is the Boltzmann constant in one of the compatible unities of energy/(mol·Kelvin).

We used the AMBER force field (ff99SB) to attribute atomic charges both at the H++ and PDB2PQR/PBSA calculations. Other PBSA parameters from APBS remained unchanged. The PBSA maps generated by the grid files were analyzed as images at the VMD software (Humphrey *et al.*, 1996). To analyze the electrostatic distribution in a comparative approach, the electrostatic maps were superposed at the respective structures using a red, white, blue color scale in the range -10.00 $k_B{\cdot}T/\beta$, -5.00 $k_B{\cdot}T/\beta$, 0.00 $k_B{\cdot}T/\beta$ for the surface electrostatic potential in all the systems. This scale was arbitrarily chosen to highlight the differences between negatively charged and neutral substituents, due to the nature of the more critical contact substitution found in this case study.

Furthermore, we performed for each mutant a short conjugate gradient (CGd) minimization protocol (Ponder and Richards, 1987), followed by inspection of the recovered vibrational eigenvectors around the minimum by principal component analysis (PCA) (Kalescky *et al.*, 2016; David and Jacobs, 2014; Sutton, 1992). The CGd method for energy minimization is efficient at finding the closest and deepest local minima of a starting medium-sized protein structure, converging in a few hundred steps (Ponder and Richards, 1987; Wako and Gō, 1987). After convergence, it is expected that the atomic coordinates oscillate around the walls of these minima, in a harmonic-like way, being both the Hessian matrix for the energies, as the covariance matrix for the coordinates around the same minima commonly used in the estimation of vibrational properties on a set of different approaches (Hinsen, 2006; Andricioaei and Karplus, 2001). In this way, we have used the PCA estimated eigenvectors for the covariance matrix of the Cα atoms for the last 8,750 steps of a 10,000 steps CGd minimization to get insights about the vibrational influences at the loop A for the contacts between residues in the positions 41 and 57 in Bgl1A and Bgl1B.

For the energy minimizations, each model was solvated with a cubic water box (TIP3P model) with a 12 Å padding using the tleap program from the AmberTools16 package (D.A. Case *et al.*, 2018, 16). The net charge of each system was properly neutralized by adding 21, 22, 25, and 24 Na^+ ions for (i) Bgl1A, (ii) H57D mutant (Bgl1A), (iii) Bgl1B, and (iv) D57H mutant (Bgl1B), respectively. Parameters for the protein, water, and ions for the four systems were all taken from the ff99SB AMBER force field (Lindorff-Larsen *et al.*, 2010). We performed the minimization protocol using the NAMD 2.10 software (Phillips *et al.*, 2005), at 300 K temperature and 1 atm pressure, with a 10Å cutoff for the non bonded interactions, periodic boundary conditions, and particle mesh Ewald treatment for the long-range electrostatic. Also, we maintained rigid hydrogen atoms, whose positions were solved step by step using the shake algorithm (Bailey and Lowe, 2009). The minimization was carried out at

a three steps procedure of CGd method with gradual system freedom. First, a 300 steps minimization was carried with a 1 kcal·mol^{-1} harmonic restraint with exponent 2 for all the heavy atoms. Then, a new 300 steps minimization was carried with harmonic restraint just for the side chains. Lastly, a productive 10,000 steps minimization was carried without restraints for the entire system, saving the coordinates at every 250 steps (hence, 40 structures per system). From these 40 frames, the last 35 were taken to structure alignment considering the protein backbone heavy atoms (Cα, C, O, and N). We calculated the average structure for the protein considering all the 35 frames and following a PCA estimation considering the Cα atoms of the region of the loop A immediately influenced by the contact substitution analyzed (residues 39 to 59) using the WORDOM package (Seeber *et al.*, 2011).

Figure S1. Vibrational mobility pattern recovered by minimization and PCA for loop A of Bgl1A, Bgl1B, and mutants.

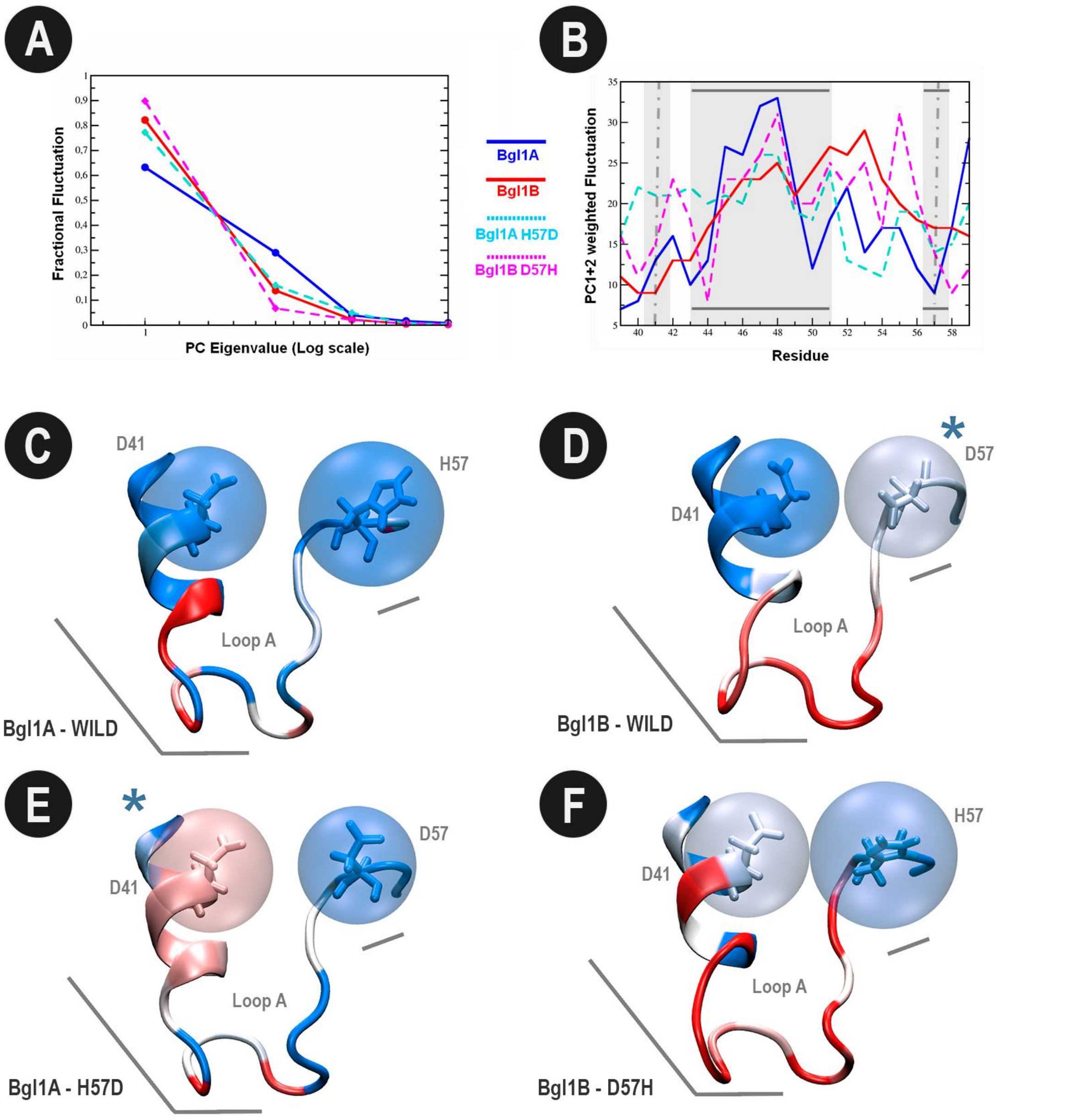

(A) Fractional representativity at the eigenvalue of the five first PCA eigenvectors for each protein; (B) fluctuation depicted at the two principal eigenvectors (PC1+2) distributed along the loop A extension from residues 39 to 59. We highlighted the mutated residues (41 and 57) and also the most affected residues (positions 43-51); (A-B) Bgl1A: blue line; Bgl1B: red line; H57D (Bgl1A): dashed cyan line; D57H (Bgl1B) dashed magenta line; (C) Bgl1A (wild); (D) Bgl1B (wild); (E) H57D (Bgl1A's mutant); (F) D57H (Bgl1B's mutant); (C-F) structural

representation of the two principal components (PC1+2). Fluctuations around the inferior, the average, and the superior standard deviation are shown in a blue-white-red color scale, respectively. Lines point to the same regions highlighted in (B). The residues 41 and 57 are shown as sticks. Transparent spheres depict the rotational influence zone; (D-E) the "*" highlights the most mobile acid residue.

Table S1. Atoms classes for each type of contact. Obtained from (Sobolev et al., 1999; Fassio et al., 2019).

Type	Amino acids (atoms)
**Hydrophobic**	ALA (CB), ARG (CB), ARG (CG), ASN (CB), ASP (CB), CYS (CB), GLN (CB), GLN (CG), GLU (CB), GLU (CG), HIS (CB), ILE (CB), ILE (CG1), ILE (CG2), ILE (CD1), LEU (CB), LEU (CG), LEU (CD1), LEU (CD2), LYS (CB), LYS (CG), LYS (CD), MET (CB), MET (CG), MET (CE), PHE (CB), PHE (CG), PHE (CD1), PHE (CD2), PHE (CE1), PHE (CE2), PHE (CZ), PRO (CB), PRO (CG), THR (CG2), TRP (CB), TRP (CG), TRP (CD2), TRP (CE3), TRP (CZ2), TRP (CZ3), TRP (CH2), TYR (CB), TYR (CG), TYR (CD1), TYR (CD2), TYR (CE1), TYR (CE2), VAL (CB), VAL (CG1), VAL (CG2)
**Positively ionizable**	ARG (NE), ARG (CZ), ARG (NH1), ARG (NH2), HIS (ND1)*, HIS (NE2)*, LYS (NZ)
**Negatively ionizable**	ASP (OD1), ASP (OD2), GLU (OE1), GLU (OE2)
**Acceptors**	ALA (O), ARG (O), ASN (O), ASN (OD1), ASP (O), ASP (OD1), ASP (OD2), CYS (O), CYS (SG), GLN (O), GLN (OE1), GLU (O), GLU (OE1), GLU (OE2), GLY (O), HIS (O), HIS (ND1), HIS (NE2), ILE (O), LEU (O), LYS (O), MET (O), MET (SD), PHE (O), PRO (O), SER (O), SER (OG), THR (O), THR (OG1), TRP (O), TYR (O), TYR (OH), VAL (O)
**Donors**	ALA (N), ARG (N), ARG (NE), ARG (NH1), ARG (NH2), ASN (N), ASN (ND2), ASP (N), CYS (N), CYS (SG), GLN (N), GLN (NE2), GLU (N), GLY (N), HIS (N), HIS (ND1), HIS (NE2), ILE (N), LEU (N), LYS (N), LYS (NZ), MET (N), PHE (N), SER (N), SER (OG), THR (N), THR (OG1), TRP (N), TRP (NE1), TYR (N), TYR (OH), VAL (N)
**Aromatic**	HIS (ring centroid), PHE (ring centroid), TRP (ring centroid), TYR (ring centroid)
**Disulfide bonds**	CYS (S)

* Histidine is unlikely to be positively charged if it is close to another basic amino acid. We assume that histidine is charged because most analyzes are done in a static way and we aim to contemplate as many cases as possible.

Table S2. Analogous contacts between proteins with PDB entries 1a6m and 1dlw.

Residues				Contact type		AVD score
1a6m (R1)	1a6m (R2)	1dlw (R1)	1dlw (R2)	1a6m R1-R2	1dlw R1-R2	
L104	S108	F78	I82	HB	HY	1.94
H36	E38	D26	T28	AT	HB	1.97
E38	Y103	T28	Q77	HY	HB	1.07
S108	R139	I82	E107	HB	HY	1.69
K102	F106	A76	T80	HY	HB	1.09
E105	R139	T79	R110	AT	HB	1.4
L9	A127	L98	Q101	HY	HB	1.6
R31	A110	N22	H84	HY	HB	1.87
V21	V66	Q13	N43	HY	HB	1.9
P100	Y103	S74	Q77	HY	HB	1.06
L89	H93	L64	H68	HB	HB	0.82
V17	L69	V12	T16	HY	HB	1.43
F43	H64	F33	Q41	AS	HY	1.3

R1: residue 1; R2: residue 2; dist.: distance between amino acid residue number; HY: hydrophobic; HB: hydrogen bonds; AT: attractive interaction; AS: aromatic stacking. Here, we do not show conserved hydrophobic contact matches.

Table S3. 1A6M Contacts (Chain A).

R1: residue 1; R2: residue 2; A1: atom 1; A2: atom 2; dist.: distance between amino acid residue number; Hy: hydrophobic; Hb: hydrogen bonds; At: attractive interaction; As: aromatic stacking; Re: repulsive interaction.

R1	R2	A1	A2	dist	Contact types
L2	W7	CB	CD1	3.8	Hy
L2	W7	CB	CE2	3.57	Hy
S3	E6	N	OE1	2.89	Hb
W7	K79	CD2	CB	3.72	Hy
W7	K79	CE2	CB	3.67	Hy
H12	K16	ND1	NZ	5.78	Re
H12	K16	NE2	NZ	4.59	Re
H12	D122	ND1	OD1	4.97	At
H12	D122	NE2	OD1	2.88	At Hb
H12	D122	NE2	OD2	4.29	At
V13	M131	CG1	CE	3.73	Hy
W14	E18	CD1	CG	3.77	Hy
W14	L76	CE3	CD1	3.69	Hy
K16	H119	CB	CE1	3.62	Hy
K16	D122	NZ	OD1	4.93	At
K16	D122	NZ	OD2	4.05	At
V17	H24	CG2	CD2	3.8	Hy
E18	K77	OE1	NZ	2.92	At Hb
E18	K77	OE2	NZ	2.77	At Hb
D20	G23	OD1	N	2.97	Hb
D20	H24	O	ND1	2.65	Hb
D20	H24	OD1	ND1	5.07	At
D20	H24	OD2	ND1	4.49	At
D20	H24	OD2	NE2	5.99	At
A22	V66	CB	CG2	3.64	Hy
H24	L69	CB	CD1	3.62	Hy
H24	H119	CG	CG	4.9	As
H24	H119	ND1	NE2	4.67	Re
H24	H119	NE2	ND1	4.67	Re

H24	H119	NE2	NE2	2.64	Re
D27	V114	CB	CG2	3.78	Hy
I28	V114	CG1	CB	3.72	Hy
I28	V114	CG1	CG2	3.72	Hy
I30	K34	CG2	CD	3.74	Hy
I30	M55	CG1	CB	3.73	Hy
R31	S35	O	OG	3.15	Hb
L32	F106	CD1	CB	3.62	Hy
F33	L40	CD2	CD1	3.77	Hy
F33	F43	CG	CG	5.48	As
F33	F43	CE1	CE1	3.63	Hy As
K34	E52	NZ	OE1	2.46	At Hb
K34	E52	NZ	OE2	4.31	At
H36	E38	ND1	OE1	4.67	At
H36	E38	ND1	OE2	4.36	At
H36	T39	O	OG1	2.73	Hb
H36	F106	CG	CG	3.63	Hy As
H36	F106	CG	CG	3.7	As
H36	F106	CG	CD2	3.63	Hy As
H36	F106	CD2	CB	3.64	Hy
H36	F106	CD2	CG	3.69	Hy As
H36	F106	CE1	CG	3.76	Hy As
H36	F106	CE1	CD1	3.39	Hy As
H36	F106	CE1	CE1	3.6	Hy As
E38	Y103	CG	CZ	3.57	Hy
L40	F46	CD1	CB	3.77	Hy
K42	K98	NZ	O	2.76	Hb
K42	Y103	CG	CE2	3.76	Hy
F43	F46	CB	CD2	3.65	Hy
F43	F46	CG	CG	5.49	As
F43	F46	CG	CD2	3.69	Hy As
F43	F46	CD2	CD2	3.59	Hy As
R45	D60	NE	OD2	2.73	Hb
R45	D60	NH1	OD1	5.01	At

R45	D60	NH1	OD2	4.8	At
R45	D60	NH2	OD1	2.95	At Hb
R45	D60	NH2	OD2	3.38	At
K47	K50	O	NZ	2.95	Hb
K50	E54	N	OE1	2.75	Hb
E59	K62	OE1	NZ	4.71	At
E59	K62	OE2	NZ	5.01	At
V66	T70	O	OG1	2.71	Hb
L72	I111	CD2	CD1	3.69	Hy
I75	L86	CD1	CD2	3.48	Hy
K78	E85	NZ	OE1	4.11	At
K78	E85	NZ	OE2	3.2	At Hb
H81	E83	ND1	OE1	5.72	At
H81	E83	ND1	OE2	4.42	At
H82	L86	CD2	CD1	3.45	Hy
H82	D141	ND1	OD2	4.79	At
H82	D141	NE2	OD1	4.84	At
H82	D141	NE2	OD2	2.69	At Hb
E83	E85	OE1	OE1	5.0	Re
L86	D141	CD1	CB	3.51	Hy
L89	H93	O	ND1	2.94	Hb
L89	H93	CD1	CE1	3.34	Hy
A90	K145	CB	CB	3.69	Hy
Q91	T95	O	OG1	2.87	Hb
S92	H97	O	N	2.79	Hb
H93	H97	CG	CG	4.83	As
H93	H97	ND1	ND1	5.84	Re
H93	H97	ND1	NE2	4.35	Re
H93	H97	CE1	CD2	3.69	Hy As
H93	H97	NE2	ND1	5.95	Re
H93	H97	NE2	NE2	4.7	Re
H93	K98	O	N	2.95	Hb
H93	I99	O	N	2.81	Hb
T95	K98	O	NZ	2.54	Hb
H97	I99	CB	CG1	3.72	Hy

I99	Y103	CG2	CB	3.77	Hy
P100	Y103	CD	CD2	3.74	Hy
K102	E105	NZ	OE1	2.77	At Hb
K102	E105	NZ	OE2	3.86	At
K102	F106	CG	CZ	3.56	Hy
Y103	F106	CG	CG	5.88	As
L104	S108	O	OG	2.85	Hb
E105	R139	OE1	NH2	5.4	At
E105	R139	OE2	NH2	4.92	At
H119	D122	ND1	OD2	5.97	At
F123	M131	CE1	CB	3.75	Hy
K145	E148	NZ	OE1	4.26	At
Y146	Y151	O	N	2.94	Hb

Table S4. 1DLW Contacts (Chain A).

R1: residue 1; R2: residue 2; A1: atom 1; A2: atom 2; dist.: distance between amino acid residue number; Hy: hydrophobic; Hb: hydrogen bonds; At: attractive interaction; As: aromatic stacking; Re: repulsive interaction.

R1	R2	A1	A2	dist	Contact types
L2	L98	CD2	CD1	3.76	Hy
F3	G8	O	N	3.05	Hb
F3	C50	CE1	CB	3.79	Hy
F3	L53	N	O	3.02	Hb
F3	P56	CE2	CD	3.79	Hy
Q5	L98	CB	CD2	3.74	Hy
L6	L53	CD1	CD1	3.71	Hy
L6	V94	CD2	CG1	3.8	Hy
L6	L98	CD2	CD2	3.74	Hy
V12	T16	O	OG1	2.66	Hb
Q13	N43	NE2	OD1	2.86	Hb
V15	L89	CG1	CD2	3.69	Hy
F19	I23	CE1	CD1	3.6	Hy
F19	I23	CZ	CD1	3.61	Hy
F19	V81	CE1	CG1	3.47	Hy
F19	V81	CZ	CG1	3.5	Hy
F19	L85	CD1	CB	3.71	Hy
Y20	I23	CD2	CD1	3.71	Hy
Y20	F33	CG	CG	5.6	As
Y20	F33	CE2	CE2	3.78	Hy As
Y20	F33	CE2	CZ	3.79	Hy As
Y20	M38	CD2	CE	3.67	Hy
N22	H84	CB	CB	3.75	Hy
N22	H84	CB	CG	3.74	Hy
I23	F33	CG2	CD1	3.77	Hy
I23	F33	CG2	CE1	3.65	Hy
D26	T28	OD1	N	2.76	Hb
D26	T28	OD1	OG1	3.02	Hb
D26	T28	OD2	OG1	3.09	Hb
D26	H84	CB	CE1	3.8	Hy

D26	H84	OD1	NE2	4.98	At
D26	H84	OD2	ND1	5.03	At
D26	H84	OD2	NE2	3.11	At Hb
I36	Q41	CG2	CG	3.71	Hy
D37	N40	OD1	N	2.93	Hb
P39	N43	O	ND2	3.2	Hb
Q41	T45	O	OG1	2.9	Hb
K44	R62	NZ	NH1	4.83	Re
F48	W59	CG	CG	5.04	As
F48	W59	CD1	CG	3.78	Hy As
F48	W59	CD1	CD2	3.71	Hy As
F48	W59	CD1	CE2	3.76	Hy As
F48	L64	CE1	CD1	3.49	Hy
F48	L64	CZ	CD1	3.53	Hy
F48	V109	CE2	CG1	3.78	Hy
F48	V109	CZ	CG1	3.68	Hy
C50	G55	O	N	2.82	Hb
A52	V105	CB	CG1	3.57	Hy
W59	R62	NE1	O	2.97	Hb
R62	V67	CB	CG2	3.5	Hy
N63	K65	OD1	N	3.04	Hb
N63	E66	OD1	N	3.1	Hb
L64	H68	O	ND1	2.8	Hb
K65	D112	NZ	OD1	4.87	At
H68	V113	CE1	CG2	3.79	Hy
V73	T115	O	N	2.96	Hb
V73	V116	O	N	3.15	Hb
S74	Q77	N	OE1	3.04	Hb
N75	T79	O	OG1	3.07	Hb
N75	R110	OD1	NH1	3.05	Hb
A76	T80	O	OG1	3.12	Hb
F78	I82	CE2	CG1	3.5	Hy
F78	I82	CE2	CD1	3.76	Hy
F78	V109	CZ	CG2	3.75	Hy
F78	R110	CE2	CB	3.72	Hy

F78	V114	CB	CG2	3.38	Hy
I82	V103	CG2	CG1	3.56	Hy
H84	S87	O	OG	3.1	Hb
L85	A106	CD2	CB	3.65	Hy
R86	T90	O	OG1	2.73	Hb
R86	E100	NE	OE2	3.01	Hb
R86	E100	NH1	OE1	5.32	At
R86	E100	NH1	OE2	5.16	At
R86	E100	NH2	OE1	3.25	At
R86	E100	NH2	OE2	3.85	At
R86	E107	NH2	OE2	5.33	At
L89	T102	CD1	CG2	3.79	Hy
V94	L98	CG1	CB	3.68	Hy
V94	L98	CG1	CG	3.59	Hy
V94	L98	CG1	CD2	3.6	Hy
L98	T102	O	OG1	2.77	Hb
V105	T108	O	OG1	2.78	Hb
E107	R110	OE1	NH1	4.92	At
E107	R110	OE1	NH2	2.98	At Hb
E107	R110	OE2	NH2	3.89	At
R110	V114	CG	CG2	3.77	Hy

Table S5. Models Assessment (BGL1B).

Results for the homology modelling project "BGA1B" submitted to SWISS-MODEL workspace on May 12, 2020, 11:58 p.m.. The SWISS-MODEL template library (SMTL version 2020-05-06, PDB release 2020-05-01) was searched with BLAST (Camacho et al.) and HHBlits (Remmert et al.) for evolutionary related structures matching the target sequence. Overall 784 templates were found. Template search with BLAST and HHBlits has been performed against the SWISS-MODEL template library (SMTL, last update: 2020-05-06, last included PDB release: 2020-05-01). The target sequence was searched with BLAST against the primary amino acid sequence contained in the SMTL. A total of 138 templates were found. An initial HHblits profile has been built using the procedure outlined in (Remmert et al.), followed by 1 iteration of HHblits against NR20. The obtained profile has then been searched against all profiles of the SMTL. A total of 646 templates were found. For each identified template, the template's quality has been predicted from features of the target-template alignment. The templates with the highest quality have then been selected for model building. Models are built based on the target-template alignment using ProMod3. Coordinates which are conserved between the target and the template are copied from the template to the model. Insertions and deletions are remodelled using a fragment library. Side chains are then rebuilt. Finally, the geometry of the resulting model is regularized by using a force field. In case loop modelling with ProMod3 fails, an alternative model is built with PROMOD-II (Guex et al.). The global and per-residue model quality has been assessed using the QMEAN scoring function (Studer et al.). The quaternary structure annotation of the template is used to model the target sequence in its oligomeric form. The method (Bertoni et al.) is based on a supervised machine learning algorithm, Support Vector Machines (SVM), which combines interface conservation, structural clustering, and other template features to provide a quaternary structure quality estimate (QSQE). The QSQE score is a number between 0 and 1, reflecting the expected accuracy of the interchain contacts for a model built based on a given alignment and template. Higher numbers indicate higher reliability. This complements the GMQE score which estimates the accuracy of the tertiary structure of the resulting model.

Model selected

Model #02	File	Built with	Oligo-State	Ligands	GMQE	QMEAN
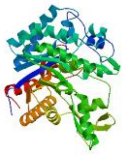	PDB	ProMod3 3.0.0	monomer	None	0.76	-1.89

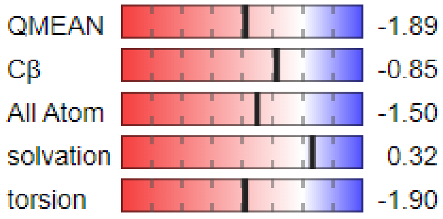

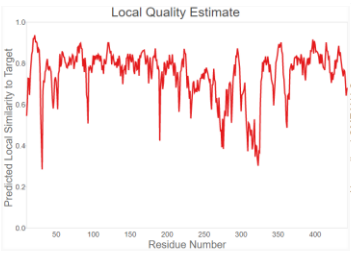

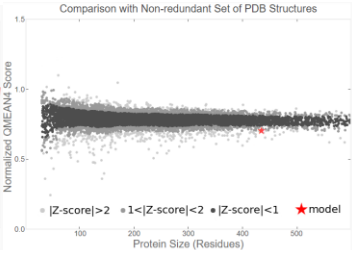

Template

**Template**	**Seq Identity**	**Oligo-state**	**QSQE**	**Found by**	**Method**	**Resolution**	**Seq Similarity**	**Range**	**Coverage**	**Description**

4gxp.1.A	43.09	monomer	0.00	BLAST	X-ray	3.00Å	0.42	11 - 445	0.97	Beta-glucosidase Chimeric protein
**Alignment**										
Target MTKLTLPYDSKMFDEDFIFGVATSSFQIEGA--RETRLDCIWDTFCAQENTISDRSNGDVACDHIAHWQQDIQLICDLGV<br>4gxp.1.A ----------KKFPEGFLWGVATASYQIEGSPLADGAGMSIWHTFSHTPGNVKNGDTGDVACDHYNRWKEDIEIIEKLGV<br><br>Target DAYRFSISWPRVM-HADGTLNETGLAFYIELIDALKAKGKKIFVTMYHWDLPQYLEDEGGWLNRDTAYAFAQYCDLVSQR<br>4gxp.1.A KAYRFSISWPRILPEGTGRVNQKGLDFYNRIIDTLLEKGITPFVTIFHWDLPFALQLKGGLLNREIADWFAEYSRVLFEN<br><br>Target IGDKVDAYTTLNEPFCAGYLSYEMGVHAPGLTGRKNGRQASHHLLLAHGLAMQVLRKNCPNADVGIVINVHPGYALTDSA<br>4gxp.1.A FGDRVKNWITFNEPLCSAIPGYGSGTFAPGRQSTSEPWTVGHNILVAHGRAVKVFRETVKDGKIGIVLNGDFTYPW-DAA<br><br>Target E--DIEATKMGTDYLFHWYIDPLLKQSYPSVMDKLSLEERPDILEGDMALIAQPLDFIGMNYYTRNVYK------MGDD-<br>4gxp.1.A DPADKEAAERRLEFFTAWFADPIYLGDYPASMRKQLGDRLPTFTPEERALVHGSNDFYGMNHYTSNYIRHRSSPASADDT<br><br>Target -GWFEIV-TPEPGNL----TEMGW-EIVPEAMTKMLIELDQQYDLPPMYITENGAAMPD-VRQGNRIADQNRIDYFQSHF<br>4gxp.1.A VGNVDVLFTNKQGNCIGPETAMPWLRPCAAGFRDFLVWISKRYGYPPIYVTENGAAFDDVVSEDGRVHDQNRIDYLKAYI<br><br>Target VAVEAAMEA-GVNIKGYFAWSLMDNFEWALGYSKRFGLIYIDYETQERVWKDSAIAYKNMLASRALVTHE<br>4gxp.1.A GAMVTAVELDGVNVKGYFVWSLLDNFEWAEGYSKRFGIVYVDYSTQKRIVKDSGYWYSNVVKNNGL----										
**Ramachandran plot**										

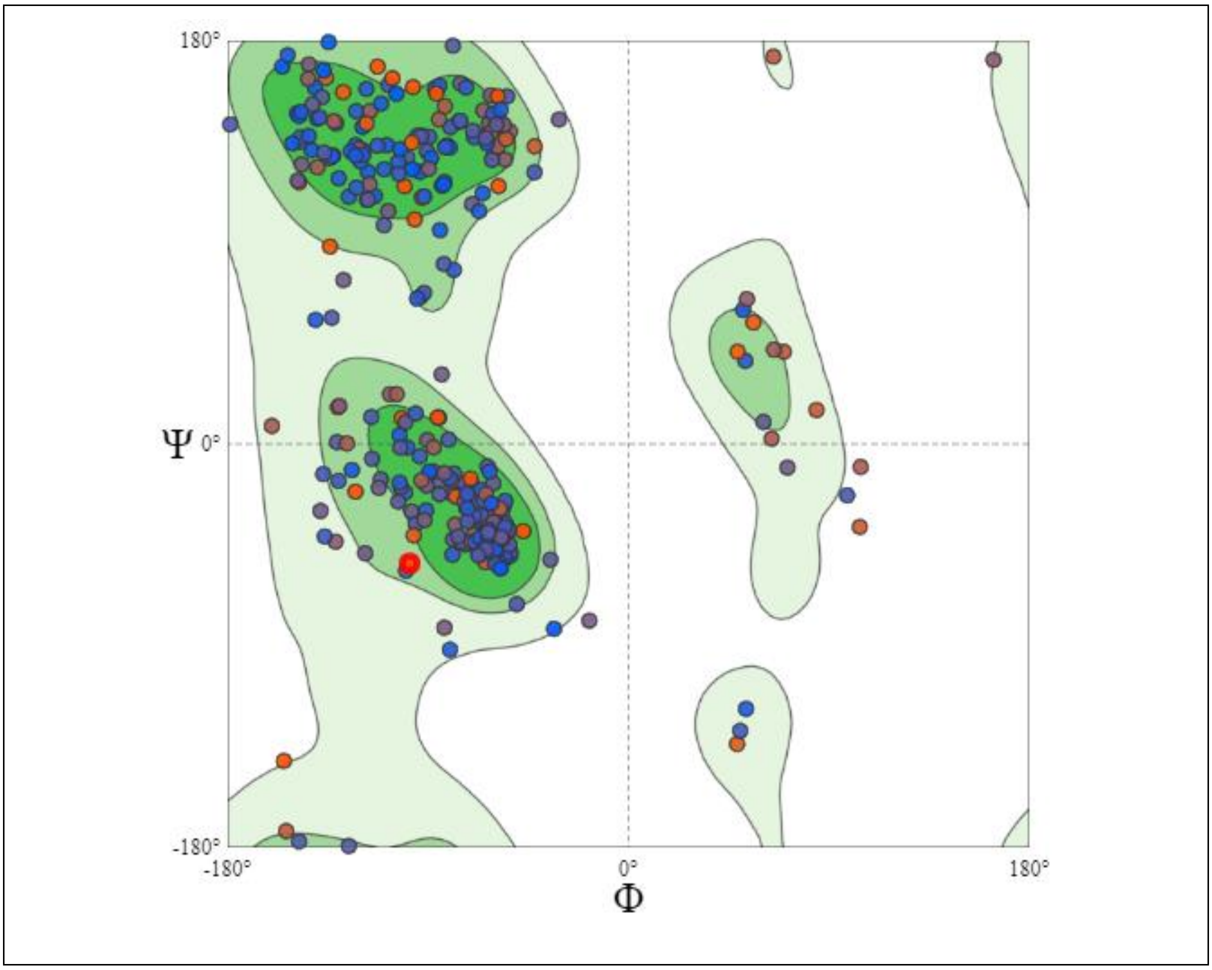
180°
Ψ 0°
-180°
-180°
0°
180°
Φ

Table S6. Models Assessment (BGL1A)

Results for the homology modelling project "BGL1A" submitted to SWISS-MODEL workspace on May 12, 2020, 11:58 p.m.. The SWISS-MODEL template library (SMTL version 2020-05-06, PDB release 2020-05-01) was searched with BLAST (Camacho et al.) and HHBlits (Remmert et al.) for evolutionary related structures matching the target sequence. Overall 786 templates were found (Table T2).

Model Selected

Model #02	File	Built with	Oligo-State	Ligands	GMQE	QMEAN
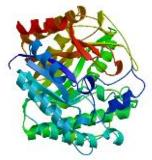	PDB	ProMod3 3.0.0	monomer	None	0.75	-2.61

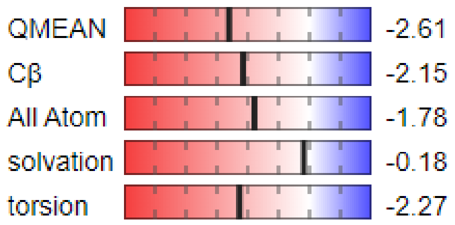

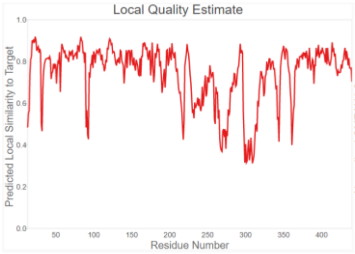

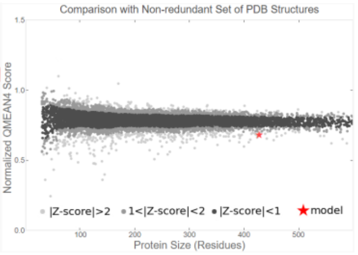

Template

Template	Seq Identity	Oligo-state	QSQE	Found by	Method	Resolution	Seq Similarity	Range	Coverage	Description
1np2.1.A	47.00	monomer	0.00	BLAST	X-ray	2.40Å	0.43	14 - 441	0.94	beta-glycosidase

Alignment

Target MTKISLPTCSPLLTKEFIYGVATASFQIEGGSAH--RLPCIWDTFCDTPGKIADNSNGHVACDHYNNWKQDIDLIESLGV
1np2.1.A -------------AEKFLWGVATSAYQIEGATQEDGRGPSIWDTFARRPGAIRDGSTGEPACDHYHRYEEDIALMQSLGV

Target DAYRLSISWPRVITKS-GELNPEGVKFYTDILDELKKRNIKAFVTLYHWDLPQHLEDEGGWLNRETAYAFAHYVDLITLA
1np2.1.A GVYRFSVAWPRILPEGRGRINPKGLAFYDRLVDRLLAAGITPFLTLYHWDLPQALEDRGGWRSRETAFAFAEYAEAVARA

Target FGDRVHSYATLNEPFCSAFLGYEIGIHAPGKVGKQYGRKAAHHLLLAHGLAMTVLKQNSPTTLNGIVLNFTPCYSISEDA
1np2.1.A LADRVPFFATLNEPWCSAFLGHWTGEHAPGLRNLEAALRAAHHLLLGHGLAVEALRAAGARRV-GIVLNFAPAYG-----

Target DDIAATAFADDYLNQWYMKPIMDGTYPAIIEQLPSAHLPDIHDGDMAIISQSIDYLGINFYTRQFYKAHPTEIYEPIE--
1np2.1.A EDPEAVDVADRYHNRYFLDPILGRGYPESPFQDPPPA--PILSRDLEAIARPLDFLGVNYYAP--VRVAPGTGPLPVRYL

Target -PTGPLTDMGWEIYPKSFTELLVTLNNTYTLPPIFITENGAAMPDSY-NNGEINDVDRLDYYNSHLNAVHNATEQGVRID<br>1np2.1.A PPEGPVTAMGWEVYPEGLYHLLKRLGREVPW-PLYITENGAAYPDLWTGEAVVEDPERVAYLEAHVEAALRAREEGVDLR<br><br>Target GYFAWSLMDNFEWAEGYLKRFGIVYVDYSTQQRTIKNSGLAYKALISNR<br>1np2.1.A GYFVWSLMDNFEWAFGYTRRFGLYYVDFPSQRRIPKRSALWYRERIAR-
**Ramachandran plot**
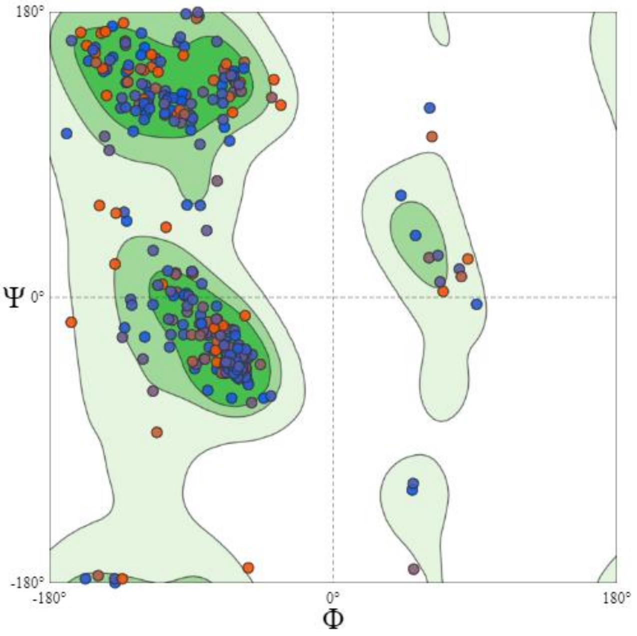

Table S7. Models Assessment (D57H__BGL1B)

Results for the homology modelling project "D57H - BGL1B" submitted to SWISS-MODEL workspace on May 13, 2020, 12:01 a.m.. The SWISS-MODEL template library (SMTL version 2020-05-06, PDB release 2020-05-01) was searched with BLAST (Camacho et al.) and HHBlits (Remmert et al.) for evolutionary related structures matching the target sequence in Table T1. For details on the template search, see Materials and Methods. Overall 790 templates were found (Table T2).

Model Selected

Model #02	File	Built with	Oligo-State	Ligands	GMQE	QMEAN
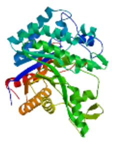	PDB	ProMod3 3.0.0	monomer	None	0.76	-2.58

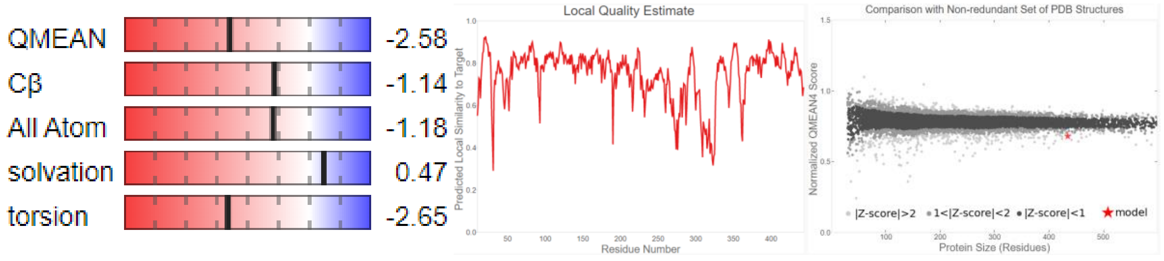

Template

**Template**	**Seq Identity**	**Oligo-state**	**QSQE**	**Found by**	**Method**	**Resolution**	**Seq Similarity**	**Range**	**Coverage**	**Description**
4gxp.1.A	42.86	monomer	0.00	BLAST	X-ray	3.00Å	0.42	11 - 445	0.97	Beta-glucosidase Chimeric protein

Alignment

Target MTKLTLPYDSKMFDEDFIFGVATSSFQIEGA--RETRLDCIWDTFCAQENTISDRSNGHVACDHIAHWQQDIQLICDLGV
4gxp.1.A ----------KKFPEGFLWGVATASYQIEGSPLADGAGMSIWHTFSHTPGNVKNGDTGDVACDHYNRWKEDIEIIEKLGV

Target DAYRFSISWPRVM-HADGTLNETGLAFYIELIDALKAKGKKIFVTMYHWDLPQYLEDEGGWLNRDTAYAFAQYCDLVSQR
4gxp.1.A KAYRFSISWPRILPEGTGRVNQKGLDFYNRIIDTLLEKGITPFVTIFHWDLPFALQLKGGLLNREIADWFAEYSRVLFEN

Target IGDKVDAYTTLNEPFCAGYLSYEMGVHAPGLTGRKNGRQASHHLLLAHGLAMQVLRKNCPNADVGIVINVHPGYALTDSA

4gxp.1.A FGDRVKNWITFNEPLCSAIPGYGSGTFAPGRQSTSEPWTVGHNILVAHGRAVKVFRETVKDGKIGIVLNGDFTYPW-DAA<br><br>Target E--DIEATKMGTDYLFHWYIDPLLKQSYPSVMDKLSLEERPDILEGDMALIAQPLDFIGMNYYTRNVYK------MGDD-<br>4gxp.1.A<br>DPADKEAAERRLEFFTAWFADPIYLGDYPASMRKQLGDRLPTFTPEERALVHGSNDFYGMNHYTSNYIRHRSSPASADDT<br>Target -GWFEIV-TPEPGNL----TEMGW-EIVPEAMTKMLIELDQQYDLPPMYITENGAAMPD-VRQGNRIADQNRIDYFQSHF<br>4gxp.1.A<br>VGNVDVLFTNKQGNCIGPETAMPWLRPCAAGFRDFLVWISKRYGYPPIYVTENGAAFDDVVSEDGRVHDQNRIDYLKAYI<br>Target VAVEAAMEA-GVNIKGYFAWSLMDNFEWALGYSKRFGLIYIDYETQERVWKDSAIAYKNMLASRALVTHE<br>4gxp.1.A GAMVTAVELDGVNVKGYFVWSLLDNFEWAEGYSKRFGIVYVDYSTQKRIVKDSGYWYSNVVKNNGL----
**Ramachandran plot**
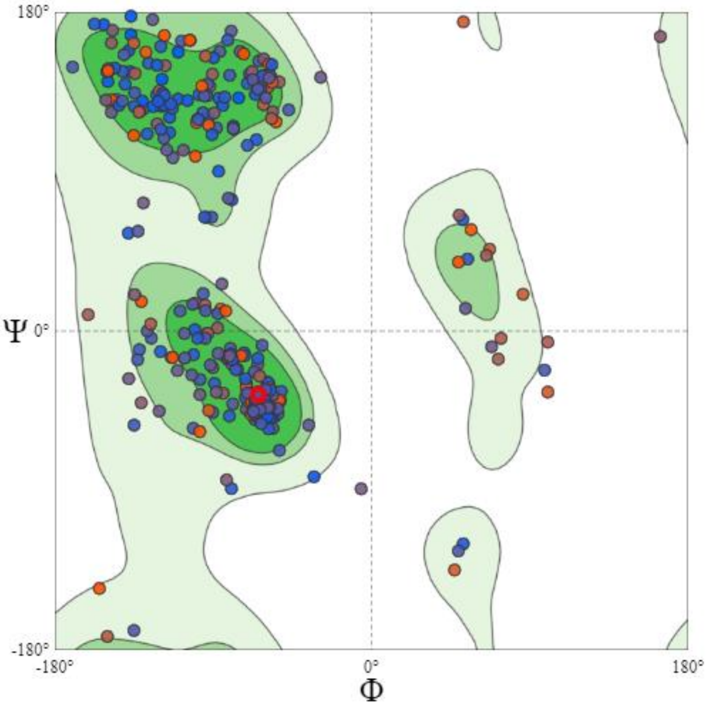

Table S8. Models Assessment (H57D_BGL1A)

Results for the homology modelling project "H57D - BGL1A" submitted to SWISS-MODEL workspace on May 13, 2020, 12:02 a.m..The SWISS-MODEL template library (SMTL version 2020-05-06, PDB release 2020-05-01) was searched with BLAST (Camacho et al.) and HHBlits (Remmert et al.) for evolutionary related structures matching the target sequence in Table T1. For details on the template search, see Materials and Methods. Overall 788 templates were found (Table T2).

**Model Selected**

Model #02	File	Built with	Oligo-State	Ligands	GMQE	QMEAN
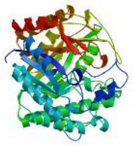	PDB	ProMod3 3.0.0	monomer	None	0.75	-2.55

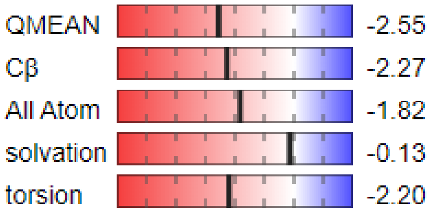

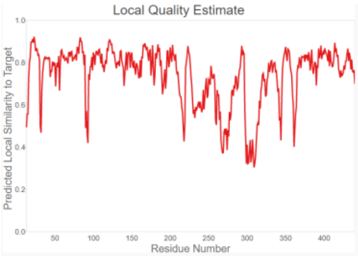

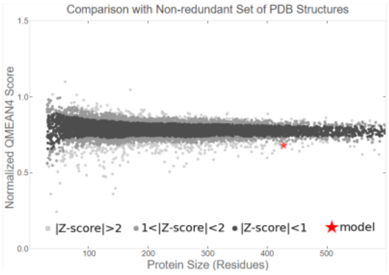

Template

Template	Seq Identity	Oligo-state	QSQE	Found by	Method	Resolution	Seq Similarity	Range	Coverage	Description
1np2.1.A	47.00	monomer	0.00	BLAST	X-ray	2.40Å	0.44	14 - 441	0.94	beta-glycosidase

Alignment

Target MTKISLPTCSPLLTKEFIYGVATASFQIEGGSAH--RLPCIWDTFCDTPGKIADNSNGDVACDHYNNWKQDIDLIESLGV
1np2.1.A -------------AEKFLWGVATSAYQIEGATQEDGRGPSIWDTFARRPGAIRDGSTGEPACDHYHRYEEDIALMQSLGV

Target DAYRLSISWPRVITKS-GELNPEGVKFYTDILDELKKRNIKAFVTLYHWDLPQHLEDEGGWLNRETAYAFAHYVDLITLA
1np2.1.A GVYRFSVAWPRILPEGRGRINPKGLAFYDRLVDRLLAAGITPFLTLYHWDLPQALEDRGGWRSRETAFAFAEYAEAVARA

Target FGDRVHSYATLNEPFCSAFLGYEIGIHAPGKVGKQYGRKAAHHLLLAHGLAMTVLKQNSPTTLNGIVLNFTPCYSISEDA
1np2.1.A LADRVPFFATLNEPWCSAFLGHWTGEHAPGLRNLEAALRAAHHLLLGHGLAVEALRAAGARRV-GIVLNFAPAYG-----

Target DDIAATAFADDYLNQWYMKPIMDGTYPAIIEQLPSAHLPDIHDGDMAIISQSIDYLGINFYTRQFYKAHPTEIYEPIE--
1np2.1.A EDPEAVDVADRYHNRYFLDPILGRGYPESPFQDPPPA--PILSRDLEAIARPLDFLGVNYYAP--VRVAPGTGPLPVRYL

Target -PTGPLTDMGWEIYPKSFTELLVTLNNTYTLPPIFITENGAAMPDSY-NNGEINDVDRLDYYNSHLNAVHNATEQGVRID
1np2.1.A PPEGPVTAMGWEVYPEGLYHLLKRLGREVPW-PLYITENGAAYPDLWTGEAVVEDPERVAYLEAHVEAALRAREEGVDLR

Target GYFAWSLMDNFEWAEGYLKRFGIVYVDYSTQQRTIKNSGLAYKALISNR<br>1np2.1.A GYFVWSLMDNFEWAFGYTRRFGLYYVDFPSQRRIPKRSALWYRERIAR-
**Ramachandran plot**
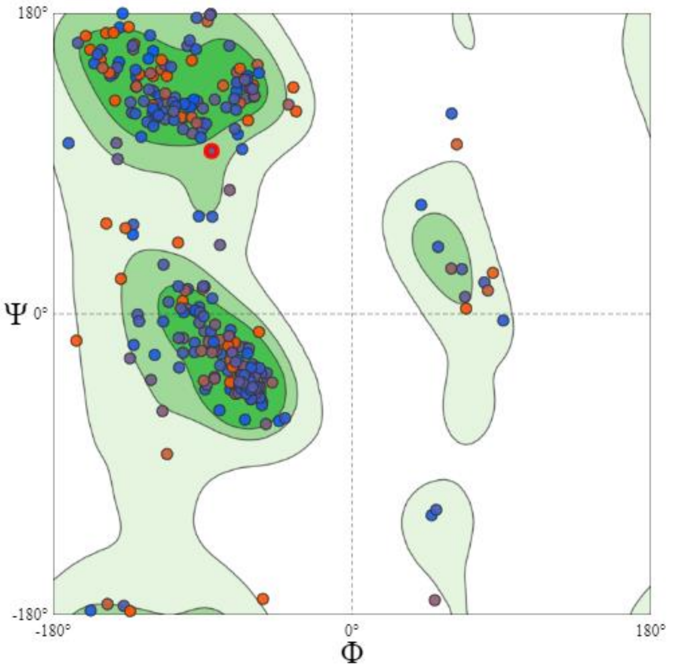

References

Anandakrishnan,R. *et al.* (2012) H++ 3.0: automating pK prediction and the preparation of biomolecular structures for atomistic molecular modeling and simulations. *Nucleic Acids Res.*, **40**, W537-541.

Andricioaei,I. and Karplus,M. (2001) On the calculation of entropy from covariance matrices of the atomic fluctuations. *J. Chem. Phys.*, **115**, 6289–6292.

Bailey,A.G. and Lowe,C.P. (2009) MILCH SHAKE: an efficient method for constraint dynamics applied to alkanes. *J. Comput. Chem.*, **30**, 2485–2493.

D.A. Case *et al.* (2018) AMBER 18 University of California, San Francisco.

David,C.C. and Jacobs,D.J. (2014) Principal Component Analysis: A Method for Determining the Essential Dynamics of Proteins. *Methods Mol. Biol. Clifton NJ*, **1084**, 193–226.

Fassio,A.V. *et al.* (2019) nAPOLI: a graph-based strategy to detect and visualize conserved protein-ligand interactions in large-scale. *IEEE/ACM Trans. Comput. Biol. Bioinform.*

Hinsen,K. (2006) Normal mode theory and harmonic potential approximations. *Norm. Mode Anal. Theory Appl. Biol. Chem. Syst. Boca Raton FL Chapman HallCRC*, 1–16.

Humphrey,W. *et al.* (1996) VMD: visual molecular dynamics. *J. Mol. Graph.*, **14**, 33–38, 27–28.

Kalescky,R. *et al.* (2016) Rigid Residue Scan Simulations Systematically Reveal Residue Entropic Roles in Protein Allostery. *PLoS Comput. Biol.*, **12**.

Lindorff-Larsen,K. *et al.* (2010) Improved side-chain torsion potentials for the Amber ff99SB protein force field. *Proteins*, **78**, 1950–1958.

Phillips,J.C. *et al.* (2005) Scalable molecular dynamics with NAMD. *J. Comput. Chem.*, **26**, 1781–1802.

Ponder,J.W. and Richards,F.M. (1987) An efficient newton-like method for molecular mechanics energy minimization of large molecules. *J. Comput. Chem.*, **8**, 1016–1024.

Seeber,M. *et al.* (2011) Wordom: A User-Friendly Program for the Analysis of Molecular Structures, Trajectories, and Free Energy Surfaces. *J. Comput. Chem.*, **32**, 1183–1194.

Sobolev,V. *et al.* (1999) Automated analysis of interatomic contacts in proteins. *Bioinformatics*, **15**, 327–332.

Sutton,A.P. (1992) Direct free energy minimization methods: application to grain boundaries. *Philos. Trans. R. Soc. Lond. Ser. Phys. Eng. Sci.*, **341**, 233–245.

Wako,H. and Gō,N. (1987) Algorithm for rapid calculation of Hessian of conformational energy function of proteins by supercomputer. *J. Comput. Chem.*, **8**, 625–635.

Waterhouse, A., Bertoni, M., Bienert, S., Studer, G., Tauriello, G., Gumienny, R., Heer, F.T., de Beer, T.A.P., Rempfer, C., Bordoli, L., Lepore, R., Schwede, T. SWISS-MODEL: homology modelling of protein structures and complexes. Nucleic Acids Res. 46(W1), W296-W303 (2018).

Guex, N., Peitsch, M.C., Schwede, T. Automated comparative protein structure modeling with SWISS-MODEL and Swiss-PdbViewer: A historical perspective. Electrophoresis 30, S162-S173 (2009).

Bienert, S., Waterhouse, A., de Beer, T.A.P., Tauriello, G., Studer, G., Bordoli, L., Schwede, T. The SWISS-MODEL Repository - new features and functionality. Nucleic Acids Res. 45, D313-D319 (2017).

Studer, G., Rempfer, C., Waterhouse, A.M., Gumienny, G., Haas, J., Schwede, T. QMEANDisCo - distance constraints applied on model quality estimation. Bioinformatics 36, 1765-1771 (2020).

Bertoni, M., Kiefer, F., Biasini, M., Bordoli, L., Schwede, T. Modeling protein quaternary structure of homo- and hetero-oligomers beyond binary interactions by homology. Scientific Reports 7 (2017).

Camacho, C., Coulouris, G., Avagyan, V., Ma, N., Papadopoulos, J., Bealer, K., Madden, T.L. BLAST+: architecture and applications. BMC Bioinformatics 10, 421-430 (2009).

Remmert, M., Biegert, A., Hauser, A., Söding, J. HHblits: lightning-fast iterative protein sequence searching by HMM-HMM alignment. Nat Methods 9, 173-175 (2012).
